# Metal-Involving Bifurcated Halogen Bonding with Iodide and Platinum(II) Center

**DOI:** 10.3390/ijms26104555

**Published:** 2025-05-09

**Authors:** Mariya A. Kryukova, Margarita B. Kostareva, Anna M. Cheranyova, Marina A. Khazanova, Anton V. Rozhkov, Daniil M. Ivanov

**Affiliations:** Institute of Chemistry, Saint Petersburg State University, 7/9 Universitetskaya Nab., Saint Petersburg 199034, Russia; m.a.kryukova@spbu.ru (M.A.K.); st097945@student.spbu.ru (M.B.K.); st063368@student.spbu.ru (A.M.C.); khazanova_m@icloud.com (M.A.K.); a.rozhkov@spbu.ru (A.V.R.)

**Keywords:** halogen bonding, bifurcate, iodide complexes, platinum(II), iodoform, molecular iodine, CP2K, periodic calculations, nitriles, dialkylcyanamides

## Abstract

The cocrystallization of *trans*-[PtI_2_(NCR)_2_] (R = NMe_2_ **1**, NEt_2_ **2**, Ph **3**, o-ClC_6_H_4_ **4**) with iodine and iodoform gave the crystalline adducts **1**∙4I_2_, **2**∙2CHI_3_, **3**∙2CHI_3_, and **4**∙4I_2_, whose structures were studied by single-crystal X-ray diffractometry (XRD). In the structures, apart from the rather predictable C–H⋯I hydrogen bonds (HBs) and I–I⋯I or C–I⋯I halogen bonds (XBs) with the iodide ligands, we identified bifurcated I–I⋯(I–Pt) and C–I⋯(I–Pt) metal-involving XBs, where the platinum center and iodide ligands function as simultaneous XB acceptors toward σ-holes of I atoms in I_2_ or CHI_3_. Appropriate density functional theory (DFT) calculations (PBE-D3/jorge-DZP-DKH with plane waves in the GAPW method) performed with periodic boundary conditions confirmed the existence of the bifurcated metal-involving I–I⋯(I–Pt) and C–I⋯(I–Pt) interactions and their noncovalent nature.

## 1. Introduction

In recent years, halogen bonding (XB) [1,2], as one of the σ-hole interactions [3], has been actively investigated in the crystal engineering of various supramolecular aggregates [4,5,6], as a tool in synthetic organometallic and coordination chemistry, [7], drug discovery [8,9], polymer science [10], and noncovalent catalysis [11,12,13,14]. XBs can also influence the stability of explosives [15,16], their photophysical properties [17,18], and their solubility [19] and volatility [20].

In a typical XB, the X⋯Nu distance tends to be shorter than the vdW radii sum, and the R–X⋯Nu angle between the covalent R–X bond and the X⋯Nu short contact tends to be linear [1]. When another halogen X’ with one neighbor R’ is a nucleophile in XB, the X⋯X’–R’ angle tends to be different from the R–X⋯Nu angle and close to 90°. This type of XBs is also known as “type II” halogen–halogen contacts [21].

Iodine-centered σ-hole XB donors, including diiodine and iodoform [22], are the most popular since iodine demonstrate the largest σ-hole and the largest polarizability among halogens, and both two properties are important in the formation of XBs [1,2]. In most cases, lone-pair-bearing non-metal atoms or electron-rich π-systems are nucleophiles in the XB formation. However, recent investigations showed that d^8^- or d^10^-centers, despite their positive charges, can also be XB acceptors due to the sterically available d_z_^2^-orbital [23]. Metal centers can also be parts of joint nucleophilic XB-accepting areas in the formation of bifurcated interactions, including two metal centers in I⋯(Rh,Rh) [24] XB, chlorides in X⋯(Cl–M) (X = Br, I; M = Pt^II^, Au^I^) [25,26,27], and even in I⋯(Cl_2_Rh_2_) interactions [24], and also carbon atoms in cyclometallated ligands in I⋯(C–M) (M = Pd^II^, Pt^II^) [28,29] bonding.

At the same time, the bifurcated metal-involving XBs, which include iodide ligands, seem to be illusive. Previously, we described the I⋯(I–Pt^II^) *possible* bifurcated XBs in [PtI_2_(COD)]∙1.5FIB (COD = 1,5-cycloocatadiene; FIB = 1,4-diiodotetrafluorobenzene) [30] and in *trans*-[PtI_2_(CN(2,6-MeC_6_H_3_))_2_]∙I_2_ [31]. In the first case, the C–I⋯Pt^II^ components of the bifurcate demonstrate I⋯Pt distances larger than the vdW sum [32] (3.7990(6) and 3.8127(4) vs. 3.73 Å), and the I–I⋯Pt angles deviate from linear (141.13(17) and 142.14(18) vs. 180°). In the second case, the I⋯Pt distances are smaller than the vdW sum (3.4600(6) and 3.4650(6) vs. 3.73 Å), but the angles strongly deviate from linear (128.10(3) and 128.27(3) vs. 180°). We believe these deviations can be explained by steric hindrance for the platinum center in the case of [PtI_2_(COD)] and the π-accepting properties [33] of both COD and isocyanide ligands, which decrease possible platinum(II) nucleophilicity.

**Figure 1:**
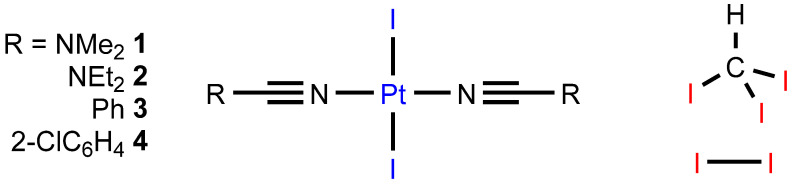
Studied XB partners. Nucleophile sites are blue, whereas electrophile sites are red.

In this work, we continued our systematic investigations of non-electrolyte iodide platinum(II) complexes as XB acceptors [19,30,34,35,36] and found bifurcated I⋯(I–Pt^II^) XBs in four cocrystals of the iodide nitrile or dialkylcyanamide complexes *trans*-[PtI_2_(NCR)_2_] (R = NMe_2_ **1**, NEt_2_ **2**, Ph **3**, o-ClC_6_H_4_ **4**) (Figure 1) with iodine and iodoform (**1**∙4I_2_, **2**∙2CHI_3_, **3**∙2CHI_3_, and **4**∙4I_2_), where both components satisfied the geometrical IUPAC criteria for XBs according to X-ray diffraction data. Further theoretical calculations confirmed the existence and noncovalent nature of the interactions.

## 2. Results and Discussion

### 2.1. Single-Crystal X-Ray Diffraction Data

**Figure 2:**
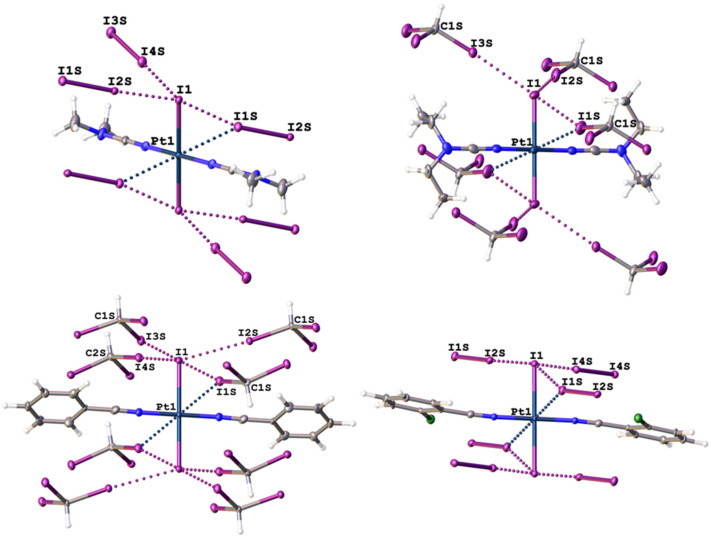
Environment of complex molecules in **1**∙4I_2_ (upper left), **2**∙2CHI_3_ (upper right), **3**∙2CHI_3_ (lower left), and **4**∙4I_2_ (lower right), where XBs are given by dotted lines. Hereinafter, thermal ellipsoids are shown with 50% probability, whereas H atoms are white, C atoms are grey, N atoms are blue, I atoms are violet, and Pt atoms are dark blue.

Complexes **1** and **4** were cocrystallized with diiodine in a 1:4 molar ratio by slow evaporation of CH_2_Cl_2_ solution, whereas **2** and **3** were cocrystallized with iodoform in a molar ratio of 1:2 after slow evaporation of their solutions in CH_3_NO_2_ or CH_2_Cl_2_/hexane (2:1, *v*:*v*), correspondingly, at RT in the dark in all cases. In both cases, the cocrystallizations gave adducts which were studied by single-crystal X-ray diffraction experiments (XRD) (Figure 2 and Figure S16, Table S1).

In all cases, the R–I⋯I–Pt (R = C, I) XBs link the iodide complex molecules with six diiodine (in **1**∙4I_2_ and **4**∙4I_2_), four iodoform (in **3**∙2CHI_3_, see Figure S16 in SI), six iodoform (in **2**∙2CHI_3_), or eight iodoform (in **3**∙2CHI_3_) molecules with I⋯I distances in the range of 3.2362(7)–3.9255(8) Å, R–I⋯I angles ranging from 154.21(13) to 178.450(17)°, and I⋯I–Pt angles ranging from 59.548(13) to 124.317(16)° (for details, see Table 1), which confirmed the XB nature of the contacts, according to the geometric IUPAC criteria [1] and their assignment to “type II” halogen–halogen contacts [21], where the metal-bound iodides act as XB acceptors.

**Figure 3:**
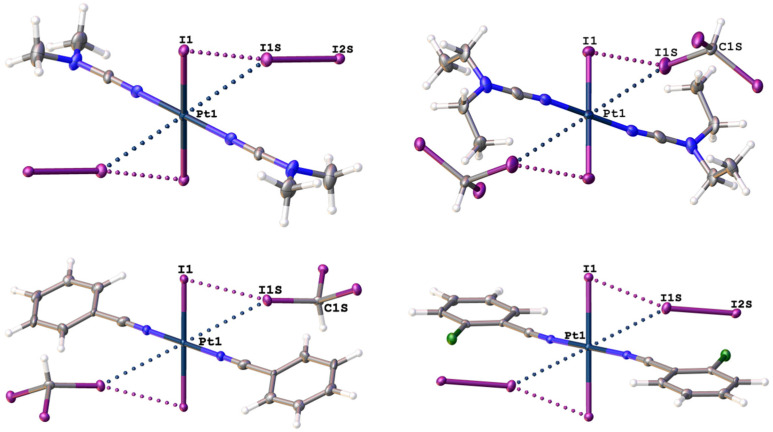
The bifurcated I⋯(I–Pt^II^) XBs in **1**∙4I_2_ (**upper left**), **2**∙2CHI_3_ (**upper right**), **3**∙2CHI_3_ (**lower left**), and **4**∙4I_2_ (**lower right**) given by dotted lines.

However, among the interactions observed in the different cocrystals, I1S⋯I1–Pt1 interactions showed the largest I⋯I distances (3.7517(5)–3.9255(8) Å), as well as the smallest R–I⋯I (154.21(13)–165.9(3)°) and I⋯I–Pt (59.548(13)–68.795(16)°) angles, which deviate from 180° and 90° for “type II” contacts, respectively. These deviations can be explained by R–I⋯Pt interactions (Table 2), which form joint R–I⋯(I–Pt) bifurcates with the latter (Figure 3), where platinum(II) and coordinated iodide centers compete with each other for the same electrophilic iodine centers in I_2_ or CHI_3_. The R–I⋯Pt components of the bifurcates demonstrate 3.4414(6)–3.7362(6) Å distances, which are shorter or close to Σ_vdW_ = 3.73 Å, and 148.77(2)–165.46(14)° angles, which are close to the same values for the R–I⋯I interactions.

Interestingly, in both I_2_ adducts, most of the intramolecular I–I distances (2.7519(8) and 2.7519(8) Å in **1**∙4I_2_ and 2.7253(5) and 2.7328(4) Å in **4**∙4I_2_) are larger than those in solid I_2_ (2.7178(1) Å) [37] but longer than those in “coordinated” triiodide (2.774(1) Å for I–I–I–Pd moiety [38]), and the bonding picture can be described as intermediate between these cases. Note that the I4S–I4S bond (2.6940(5) Å) in **4**∙4I_2_ is even shorter than that in solid I_2_ since this molecule in only involved in two moderate (2.7178(1) Å) I–I⋯I–Pt XBs.

**Figure 4:**
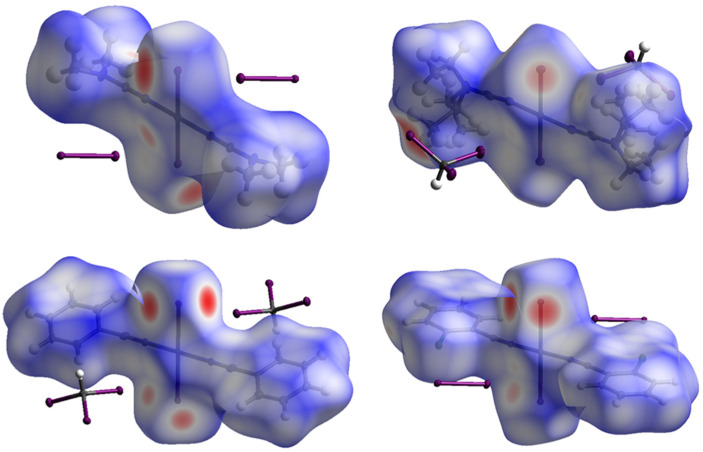
Hirshfeld surface for **1**∙4I_2_ (**upper left**), **2**∙2CHI_3_ (**upper right**), **3**∙2CHI_3_ (**lower left**), and **4**∙4I_2_ (**lower right**). Intermolecular contacts closer than the sum of Bondi vdW radii (Σ_vdW_) are highlighted in red, longer contacts are shown in blue, and contacts with a value of approximately Σ_vdW_ are shown in white.

To verify what types of noncovalent forces contribute to the crystal packing in the structures of **1**∙4I_2_, **2**∙2CHI_3_, **3**∙2CHI_3_ and **4**∙4I_2_, we carried out Hirshfeld surface analysis [39,40] using the CrystalExplorer program [41]; for visualization, we used the mapping of the normalized contact distance (*d*_norm_) (Figure 4). The Hirshfeld surface analysis indicated the domination of I⋯H type contacts in all cases (from 34.4 to 46.4%), which was expected as the fraction of the H atoms is large (Table 3). The contribution of I⋯I and Pt⋯I intermolecular contacts to the Hirshfeld surfaces was also significant and comprised 7.4–19.6% (for I⋯I) and 2.0–3.3% (for Pt⋯I), respectively. Although the contacts that involve H atoms mainly contribute to the crystal packing, I⋯I and Pt⋯I interactions are the most fascinating in the context of this work, and therefore, they are consistently discussed further.

### 2.2. Theoretical Consideration

To obtain more arguments for the existence and noncovalent nature of the interactions, we performed DFT Douglas–Kroll–Hess second-order scalar relativistic (DKH2) calculations with periodic boundary conditions using experimentally obtained atomic coordinates, PBE [42] -D3 [43,44] level of theory with jorge-DZP-DKH [45,46,47,48] basis sets, which were realized in CP2K [49,50,51,52,53,54,55] in Gaussian augmented plane wave (GAPW) [56] formalism.

In all cases, the Bader quantum theory of atoms-in-molecules (QTAIM) topological analysis of electron density ρ was used for periodic wavefunctions for all crystal models under consideration. It showed the bond critical points (BCPs) between iodine in iodoform or diiodine and the platinum atom in **1**–**4** in all cases, which correspond to the I⋯Pt components of the bifurcate XBs (Table 4). The typically noncovalent nature of the interactions was confirmed by the parameters of the corresponding BCPs, including (i) small values of ρ; (ii) positive and small values of Laplacian ∇^2^ρ; (iii) positive or close-to-zero values of energy density H; and (iv) the balance of the Lagrangian kinetic energy G and the potential energy density V [57].

For all monofurcated I⋯I interactions, the corresponding BCPs were also found. In the cases of iodoform-involving interactions, the |V|/G relation is also less than 1, and the C–I⋯I XBs can be considered purely noncovalent (Table 5). In most cases, the I–I⋯I monofurcated XBs demonstrate a non-negligible covalent character, since |V|/G > 1.

**Figure 5:**
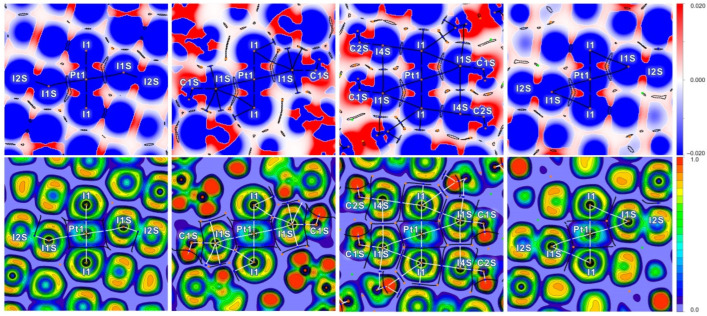
Sign(λ_2_)ρ with RDG = 0.5 dotted contour lines (**upper**) and ELF (**lower**) projections for the bifurcated I⋯(I–Pt) XBs in crystal models of **1**∙4I_2_ (**first column**), **2**∙2CHI_3_ (**second column**), **3**∙2CHI_3_ (**third column**), and **1**∙4I_2_ (**fourth column**). QTAIM white (for ELF) or black (for sign(λ_2_)ρ with RDG) bond paths, brown nuclear, blue bond, and orange ring critical points, and black (ELF) interbasin paths.

In the case of the I2S–I1S⋯I1–Pt1 component of bifurcate in **1**∙4I_2_, no BCP was detected (Figure 5, upper left). However, the projection of sign(λ_2_)ρ function in NCI analysis [58] showed the light blue area with negative λ_2_ between I1S and I1 atoms. The ρ minimum along the I1S⋯I1 line is not negligible (0.009 e/Bohr^3^) and is located in the RDG = 0.5 area, indicated by a dotted line. The parameters in this minimum can also be found in Table 5. We suppose the I2S–I1S⋯(I1–Pt1) can also be considered a true bifurcate, since for both the I1S⋯Pt1 and I1S⋯I1 components, blue areas of negative sign(λ_2_)ρ values were found. In all other cases of I⋯I components of the bifurcates, BCPs were successfully detected, and the BCP parameters are in accordance with longer I⋯I distances, and these interactions can also be treated as purely noncovalent.

In all cases, the nucleophilicity of both iodide and platinum(II) centers toward iodine in iodoform or diiodine was confirmed by electron localization function (ELF) [59,60,61] projections with topological bond paths and critical points for ρ (Figure 5). The I1S⋯Pt1 and I1S⋯I1 bond paths are far from the orange lone pair areas with high ELF values on I1S atoms in all cases. The green low-ELF area, with corresponds to the σ-hole on the I1 atom, is also directed to the yellow high-ELF area on iodide in the case of the I1S⋯I1 interaction in **1**∙4I_2_. Thus, in all cases, the I1 centers are electrophilic partners toward both iodide and platinum(II) centers.

**Figure 6:**
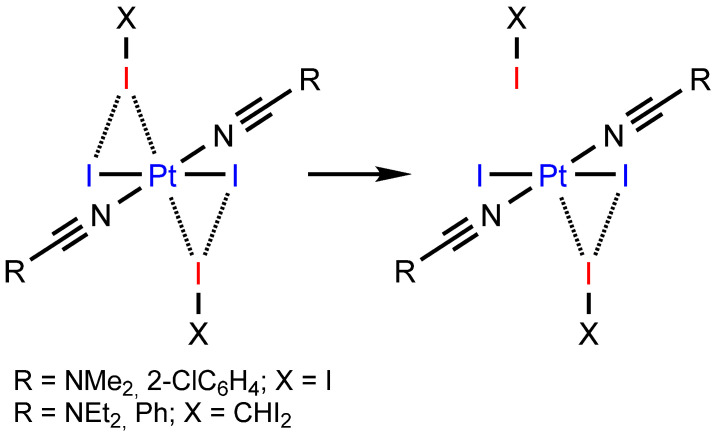
Scheme of cluster model decomposition for sSAPT0 calculations.

To evaluate the energies of the bifurcate interactions and decompose them into electrostatic, exchange, induction, and dispersion parts, we performed scaled symmetry adapted perturbation theory (sSAPT0) [62,63] calculations with def2-TZVP [64,65] bases for the dissociation of heterotrimeric clusters extracted from crystal structures to isolated iodoform or diiodine molecules and heterodimeric clusters (Figure 6 and Table 6).

According to the sSAPT0 calculations, the energy I⋯(I–Pt) bifurcates are in the range from –8.58 to –9.55 kcal/mol, with diiodine interactions being expectedly stronger. In all cases, the dispersion component is more negative than the electrostatic component, and the E_disp_/E_elst_ ratio is the largest for the **2**∙(CHI_3_)_2_ cluster, which can be explained by the largest NEt_2_ substituents and polarizable CHI_3_ molecules.

## 3. Materials and Methods

K_2_[PtCl_4_], PtI_2_, KI, I_2_, CHI_3_, acetonitrile, N,N-dimethylcyanamide, N,N-diethylcyanamide, benzonitrile, 2-chlorobenzonitrile, and all solvents were obtained from Sigma Aldrich (Merck, Germany) and used as received.

The NMR spectra were recorded on Bruker AVANCE III 400 and Bruker AVANCE 500 spectrometers (Billerica, MA, USA) at ambient temperature in acetone-d^6^ or CDCl_3_ (at 500 or 400, 126 or 101, 107 or 86 MHz for ^1^H, ^13^C{^1^H}, and ^195^Pt NMR spectra, respectively) (Figures S1–S9). K_2_[PtCl_4_] in D_2_O was used as a standard for the ^195^Pt NMR measurements. IR spectra (Figures S10–S12) were recorded on a Bruker (Billerica, MA, USA) TENSOR 27 FT-IR spectrometer (4000–400 cm^–1^, KBr or CsI pellets) (Figure S4). TLC was performed on Merck 60 F254 SiO_2_ plates (Sigma Aldrich, Merck, Germany). The HRESI^+^-MS data (Figures S13–S15) were obtained on a “MaXis”, Bruker Daltonik GmbH (Billerica, MA, USA), spectrometer equipped with an electrospray ionization (ESI) source; CH_2_Cl_2_ was used as a solvent.

### 3.1. Synthesis of Trans-[PtI_2_(NCNR_2_)_2_] (R = Me 1, Et 2) Dialkylcyanamide Complexes

Complex **1** was synthesized as previously reported [35]. A 2-fold excess of KI (0.4 g, 2.4 mmol) was added to an aqueous solution of K_2_PtCl_4_ (0.5 g, 1.2 mmol, 2.5 mL of H_2_O). The reaction mixture was left for 15 min until the darkening of the solution ceased. After that, a 10-fold excess of N,N-dimethylcyanamide (NCNMe_2_, 12 mmol, 0.876 mL) was added to the solution and the reaction mixture was left for a week until the solution became clear and an orange precipitate was formed. The resulting precipitate was filtered, washed with three portions of 3 mL of water and diethyl ether, and then dried in air at room temperature. The substance was purified by column chromatography on silica gel (Merck 60 F254, Sigma Aldrich, Merck, Germany), CH_2_Cl_2_:EtOAc = 4:1, *v*/*v*, first fraction). Yield: 66.7% (473 mg). Analytical data are in accordance with those reported earlier [35].

For **2**, the synthetic procedure was based on those previously reported for **1** and *trans*-[PtI_2_(NCN(CH_2_)_5_)_2_] [35]. The procedure for **2** was analogous to that described **1**, except that N,N-diethylcyanamide (NCNEt_2_) was used instead of NCNMe_2_. The purification steps followed the same column chromatography method, affording trans-[PtI₂(NCNEt_2_)_2_] at similarly high purity.

Yield: 77.9% (605 mg). TLC (eluent is CH_2_Cl_2_:MeOH 50:1, *v*/*v*): R_f_ = 0.79. HRESI^+^-MS (MeOH, m/z): 667.9313 ([M + Na]^+^, calcd 667.9317)). IR (CsI, selected bonds, cm^−1^): 2932 (w), 2853 (w), ν(C–H); 1451 (w), 1284 (m), δ(CH_3_); 2290 (s), ν(C≡N); 1094 (w), ν(C–N). ^1^H NMR (acetone-d^6^, δ): 3.15 (q, *J* = 7.2 Hz, 2H, NC*H*_2_), 1.18 (t, *J* = 7.4 Hz, 3H, NCH_2_C*H*_3_) ppm. ^13^C{^1^H} NMR (acetone-d^6^, δ): 119.21 (C≡N), 47.01 (N*C*H_2_), 13.32 (NCH_2_*C*H_3_) ppm. ^195^Pt NMR (acetone-d^6^, δ): −3652.11 ppm.

### 3.2. Synthesis of Trans-[PtI_2_(NCR)_2_] (R = Ph 3, 2-ClC_6_H_4_ 4) Nitrile Complexes

Complex **3** was synthesized as previously reported [66].

Complex **4** was synthesized from *cis*/*trans*-[PtCl_2_(EtCN)_2_] [67] in two steps, where all operations were performed under argon atmosphere using standard Schlenk techniques and all solvents were dried by the usual procedures and freshly distilled before use.

I. A solution of [PtCl_2_(EtCN)_2_] (211 mg, 0.56 mmol, 1 equiv) in 1.4 mL of dried toluene was prepared, and an excess of *o*-ClC_6_H_4_CN (309 mg, 2.24 mmol, 4 equiv) was added. The resulting mixture was purged with argon and heated under reflux in an oil bath for 5 h with continuous stirring. After completion of the reaction, both the nitrile EtCN and toluene were removed under reduced pressure. The resulting residue was then subjected to column chromatography on silica gel (Merck 60 F254, CH_2_Cl_2_:MeOH = 100:1, *v*/*v*), which afforded the *cis*- and *trans*- isomers of [PtCl_2_(2-ClC_6_H_4_CN)_2_] as separate fractions. Yield: 47% (142 mg). TLC (eluent is CH_2_Cl_2_:MeOH = 100:1, *v*/*v*): R_f_ = 0.52. HRESI^+^-MS (MeOH, *m*/*z*): 562.8972 ([M + Na]^+^, calcd 562.8938), 578.8686 ([M + K]^+^, calcd 578.8678). IR (KBr, selected bonds, cm^−1^): 3063(m) ν(=C–H), 2294(m) ν(C≡N); 1580(m), 1462(m), 1433(m) ν(C=CAr); 1261(m) and 1209(m) in-ring deformations; 1061(s) ν(C–Cl); 776(s), 683(s), 555(s) (C–H oop). ^1^H NMR (CDCl_3_, δ): 7.80 (m, 1H, CHAr), 7.69 (m, 1H, CHAr), 7.58 (m, 1H, CHAr), 7.47 (m, 1H, CHAr). ^13^C{^1^H} NMR (CDCl_3_, δ): 139.07, 136.42, 135.62, 130.62, 127.55, 114.17, 110.66. ^195^Pt NMR (CDCl_3_, δ): –2348.04.

II. The mixture of [PtCl_2_(2-ClC_6_H_4_CN)_2_] (100 mg, 0.18 mmol) and NaI (54 mg, 0.36 mmol) was stirred in acetone (2 mL) at 35° in the dark for 24 h. The mixture was cooled to ambient temperature, and the precipitate was filtered off and washed twice with 1 mL of acetone. From the remaining brown solution, the solvent was removed under vacuum. The crystallization of the crude product from acetone gave **4** (89 mg, 68%) as yellow crystals. TLC (eluent is CH_2_Cl_2_:hexane = 2:1, *v*/*v*): R_f_ = 0.45. HRESI^+^-MS (MeOH, m/z): 746.2744 ([M + Na]^+^, calcd 746.7651). IR (KBr, selected bonds, cm^−1^): 3445(m) ν(=C–H), 2291(m) ν(C≡N); 1582(m), 1468(m), 1433(m), ν(C=CAr); 1266(s), 1209(s) and 1160(s) in-ring deformations; 1062(s) ν(C–Cl); 761 (s), 686(s), 557(s), 546(s), 464(s) (C–H oop). ^1^H NMR (500 MHz, Acetone-d6) δ 7.92 (dd, J = 7.8, 1.6 Hz, 1H), 7.79 (ddd, J = 8.2, 7.5, 1.6 Hz, 1H), 7.73 (dd, J = 8.2, 1.2 Hz, 1H), 7.61 (td, J = 7.6, 1.2 Hz, 1H). ^13^C NMR (126 MHz, Acetone-d6) δ 135.82, 134.76, 134.44, 130.13, 128.02, 115.62, 112.89. ^195^Pt NMR (107 MHz, Acetone-d6) δ −5050.56 (s).

### 3.3. Crystallizations of the Adducts

Single crystals of **1**·4I_2_ and **4**·4I_2_ were obtained by slow evaporation of dichloromethane (CH_2_Cl_2_) solutions at room temperature. For **1**·4I_2_, 10 mg (0.017 mmol) of the platinum complex was combined with 17.2 mg (0.068 mmol) of iodine (1:4 molar ratio), whereas **4**·4I_2_ was prepared from 10 mg (0.014 mmol) of the corresponding platinum complex and 14.0 mg (0.055 mmol) of iodine, likewise maintaining a 1:4 ratio. In both cases, the solutions were left in loosely covered vials, protected from light, and suitable crystals for X-ray diffraction typically formed within a few days.

For **2**·2CHI_3_, 5 mg (0.008 mmol) of the platinum complex and 6.1 mg (0.015 mmol) of iodoform (1:2 molar ratio) were dissolved in nitromethane (CH_3_NO_2_). In the case of **3**·2CHI_3_, 10 mg (0.015 mmol) of the complex was mixed with 12.0 mg (0.031 mmol) of iodoform (1:2 ratio) in a dichloromethane/hexane solvent mixture (2:1 *v*/*v*). Both solutions were allowed to evaporate slowly in vials protected from light, yielding single crystals suitable for further structural investigations within several days.

### 3.4. X-Ray Structure Determination and Refinement

Suitable single crystals of adducts **1**∙4I_2_, **2**∙2CHI_3_, **3**∙2CHI_3_, and **4**∙4I_2_ were studied on an Xcalibur Eos diffractometer (monochromated MoK_α_ radiation, λ = 0.71073, Oxford diffraction, Wrocław, Poland). Crystals were incubated at 100 K during data collection. Using Olex2 [68], the structures were solved with the ShelXT [69] structure solution program using intrinsic phasing and refined with the ShelXL [70] refinement package using least-squares minimization. Hydrogen atoms in all structures were placed in the ideal calculated positions according to neutron diffraction statistical data [71] and refined as colliding atoms with parameters of relative isotropic displacement. The main data of crystallography and details of refinement are given in Table S1 in Supporting Information. CCDC numbers 2441327–2441330 contain all supporting structural and refinement data.

### 3.5. Computational Details

Hirshfeld surface analysis [39,40] was performed using the CrystalExplorer 21 program [41]. The contact distances (*d*_norm_), based on *R*_vdW_ [32], were mapped on the Hirshfeld surfaces. In the color scale, the negative values of *d*_norm_ were visualized by red color, indicating contacts shorter than Σ_vdW_. The values represented in white color denote the intermolecular distances close to contacts with *d*_norm_ equal to zero. Contacts longer than Σ_vdW_ with positive *d*_norm_ values were colored in blue.

Single-point DFT calculations based on experimentally determined coordinates (Tables S2–S5) with periodic boundary conditions for *crystal* (1 × 1 × 1 cell) **1**∙4I_2_, **2**∙2CHI_3_ (only one disordered Et position preserved), **3**∙2CHI_3_, and **4**∙4I_2_ models were performed in the CP2K-8.1 program [49,50,51,52,53,54,55] with 350 Ry and 50 Ry relative plane-wave cut-offs for the auxiliary grid using the PBE [42]-D3 [43,44] functional, (i) the Gaussian/augmented plane wave (GAPW) [56] with a full-electron jorge-DZP-DKH [45,46,47,48] mixed basis set with the Douglas–Kroll–Hess second-order scalar relativistic calculations requested relativistic core Hamiltonian [72,73]. We achieved 1.0 × 10^−6^ Hartree convergence for the self-consistent field cycle in the Γ-point approximation. The 0.500 r_loc_ parameter was applied for Pt atoms in full-electron calculations. Electron localization function (ELF) [59,60,61], sign(λ_2_)ρ+RDG [58] projection analyses, and Bader [57,74,75] atoms-in-molecules topological analysis of electron density (QTAIM) were performed and visualized in Multiwfn 3.8 [76].

Symmetry adapted perturbation theory [62,63] calculations (sSAPT0/def2-TZVP [64,65]) for the **1**∙(I_2_)_2_, **2**∙(CHI_3_)_2_, **3**∙(CHI_3_)_2_, and **4**∙(I_2_)_2_ models decomposition were performed in Psi4 1.9 [77].

## 4. Conclusions

In this work, we reported four cocrystals of platinum(II) iodide dialkylcyanamide or nitrile complexes with I_2_ or CHI_3_ in a 1:4 or 1:2 ratio, respectively. In all cases, the bifurcated metal-involving C–I⋯(I–Pt) or I–I⋯(I–Pt) XBs were detected by XRD together with conventional C–I⋯I or I–I⋯I XBs. Although I⋯(I–Pt) contacts were previously reported, in this work, the bifurcated interactions with both platinum(II) and iodine centers fulfilled both the distance and angle IUPAC criteria.

The existence and the nature of the interactions were confirmed by further DFT full-electron calculations with periodic boundary conditions (PBE-D3/jorge-DZP-DKH, GAWP). The periodic wavefunctions were investigated by QTAIM topological analysis, sign(λ_2_)ρ, and ELF projections, which showed the nucleophilicity of both the platinum(II) centers and the iodide ligands toward the same iodine electrophile. Although, in one case, BCP was found only for the I–I⋯Pt component of the bifurcate, the iodide-involving interaction was confirmed by the sign(λ_2_)ρ+RDG analysis, which demonstrated the area of negative sign(λ_2_)ρ value along the I⋯I line. This observation is in accordance with previously reported trifurcated C–I⋯(Cl–Pt–Cl) interactions [78], where both I⋯Cl components were also confirmed by sign(λ_2_)ρ+RDG analysis without corresponding BCPs. Thus, we believe further investigations of polycenter noncovalent interactions require sign(λ_2_)ρ+RDG analysis, also known as NCI analysis, since one large ρ maximum along the zero-flux surface, i.e., BCP, can interfere with the detection of another, smaller maximum nearby.

**Figure 7:**
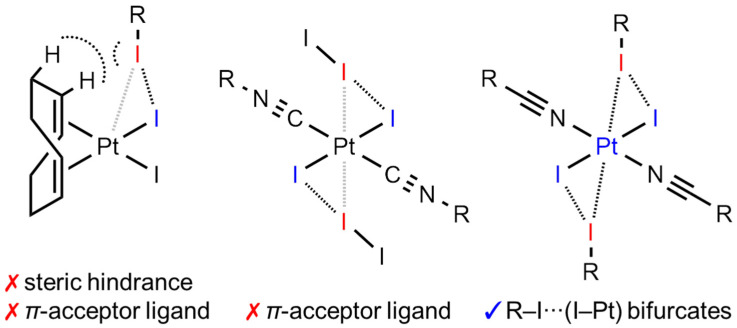
Differences between previously reported R–I⋯(I–Pt) contacts with [PtI_2_(COD)] (**left**) or *trans*-[PtI_2_(CN(2,6-MeC_6_H_3_))_2_] (**center**) and the R–I⋯(I–Pt) bifurcate XBs with *trans*-[PtI_2_(NCR)_2_] (**right**). Nucleophile sites are blue, whereas electrophile sites are red.

In all four cocrystals of platinum(II) iodide dialkylcyanamide or nitrile complexes, “true” R–I⋯(I–Pt) bifurcate XBs were formed, comparing with previously reported [PtI_2_(COD)]∙1.5FIB (COD = 1,5-cycloocatadiene; FIB = 1,4-diiodotetrafluorobenzene) [30] and *trans*-[PtI_2_(CN(2,6-MeC_6_H_3_))_2_]∙I_2_ [31] (Figure 7).

We suppose that the success of both the nitrile and dialkylcyanamide structures was achieved due to (i) the small steric hindrance for the platinum center compared with [PtI_2_(COD)] and (ii) the small or even absent [79] π-accepting properties of NCR and NCNR_2_ compared with both COD and isocyanide ligands [33], which decrease the possible platinum(II) nucleophilicity.

## Figures and Tables

**Table 1:** Parameters of R–I⋯I–Pt (R = C, I) XBs in cocrystals. For components of bifurcates, italic font is used.

Structure	Interaction	d(I⋯I), Å	R_vdW_ *	∠(R–I⋯I), °	∠(I⋯I–X), °
**1**∙4I_2_	* I1S * *⋯I1–Pt1*	* 3.9255(8) *	* 0.99 *	* 164.38(2) *	* 59.548(13) *
	I2S⋯I1–Pt1	3.2362(7)	0.82	174.39(2)	105.017(18)
	I3S⋯I1S–I2S	3.4484(8)	0.87	173.48(2)	91.355(18)
	I4S⋯I1–Pt1	3.4568(8)	0.87	177.88(2)	103.462(18)
**2**∙2CHI_3_	* I1S * *⋯I1–Pt1*	* 3.9138(8) *	* 0.99 *	* 154.21(13) *	* 63.497(13) *
	I2S⋯I1–Pt1	3.5503(9)	0.90	170.8(2)	109.053(18)
	I3S⋯I1–Pt1	3.5687(6)	0.90	173.6(2)	124.317(16)
**3**∙2CHI_3_	* I1S * *⋯I1–Pt1*	* 3.7811(8) *	* 0.95 *	* 165.9(3) *	* 68.795(16) *
	I2S⋯I1–Pt1	3.5559(8)	0.90	174.7(3)	106.531(19)
	I3S⋯I1–Pt1	3.7176(8)	0.94	167.6(3)	98.484(18)
	I4S⋯I1–Pt1	3.5675(8)	0.90	173.87(19)	98.30(2)
	I5S⋯I1A–Pt1A	3.5882(8)	0.91	173.7(3)	109.571(18)
	I6S⋯I1A–Pt1A	3.6094(8)	0.91	169.6(2)	100.947(17)
**4**∙4I_2_	* I1S * *⋯I1–Pt1*	* 3.7517(5) *	* 0.95 *	* 158.298(13) *	* 66.909(8) *
	I2S⋯I1–Pt1	3.2980(4)	0.83	177.373(15)	95.769(10)
	I3S⋯I1S–I2S	3.4385(4)	0.87	178.450(17)	99.193(13)
	I4S⋯I1–Pt1	3.4783(4)	0.88	172.041(17)	88.569(8)

* R_vdW_ = d/Σ_vdW_; Σ_vdW_(I + I) = 3.96 Å [32].

**Table 2:** Parameters of R–I⋯Pt (R = C, I) XBs in cocrystals.

Structure	Interaction	d(I⋯Pt), Å	R_vdW_ *	∠(R–I⋯Pt), °
**1**∙4I_2_	I2S–I1S⋯Pt1	3.4414(6)	0.92	148.77(2)
**2**∙2CHI_3_	C1S–I1S⋯Pt1	3.6065(7)	0.97	165.46(14)
**3**∙2CHI_3_	C1S–I1S⋯Pt1	3.7362(6)	1.00	152.8(3)
**4**∙4I_2_	I2S–I1S⋯Pt1	3.6338(3)	0.97	150.58(1)

* R_vdW_ = d/Σ_vdW_; Σ_vdW_(I + I) = 3.96 Å [32].

**Table 3:** Results of Hirshfeld surface analysis for the X-ray diffraction structures of all structures.

Structure	Contributions of Different Intermolecular Contacts to the Molecular Hirshfeld Surface *
**1**∙4I_2_	Pt⋯I 3.3%, I⋯I 19.6%, I⋯N 9.7%, I⋯C 3.8%, I⋯H 46.4%, C⋯H 1.7%, H⋯H 14.8%
**2**∙2CHI_3_	Pt⋯I 2.0%, I⋯I 7.4%, I⋯N 1.8%, I⋯H 44.5%, N⋯H 7.8%, C⋯H 2.9%, H⋯H 31.5%
**3**∙2CHI_3_	Pt⋯I 2.0%, Pt⋯H 1.6%, I⋯I 13.7%, I⋯N 5.3%, I⋯C 6.2%, I⋯H 38.6%, N⋯H 5.1%, C⋯C 3.1%, C⋯H 16.8%, H⋯H 7.0%
**4**∙4I_2_	Pt⋯I 2.8%, I⋯I 12.8%, I⋯Cl 10.1%, I⋯N 6.1%, I⋯C 2.1%, I⋯H 34.4%, Cl⋯C 8.9%, N⋯H 1.3%, C⋯C 6.9%, C⋯H 11.3%, H⋯H 2.4%

* The contributions of all other intermolecular contacts do not exceed 1%.

**Table 4:** Electron density ρ (in e/bohr^3^), Laplacian ∇^2^ρ (in e/bohr^5^), potential energy density V, Lagrangian kinetic energy G, and energy density H (in hartree/bohr^3^) at the bond critical points (3, −1), corresponding to the I⋯Pt interactions with d(I⋯Pt) (in Å) in **1**∙4I_2_, **2**∙2CHI_3_, **3**∙2CHI_3_, and **4**∙4I_2_.

Structure	Interaction	d(I···Pt)	ρ	∇^2^ρ	G	V	H	|V|/G
**1**∙4I_2_	I2S–I1S⋯Pt1	3.4414	0.018	0.039	0.010	−0.010	0.000	°1.003
**2**∙2CHI_3_	C1S–I1S⋯Pt1	3.6065	0.012	0.029	0.006	−0.006	0.000	°0.872
**3**∙2CHI_3_	C1S–I1S⋯Pt1	3.7362	0.010	0.025	0.005	−0.004	0.001	°0.798
**4**∙4I_2_	I2S–I1S⋯Pt1	3.6338	0.012	0.030	0.007	−0.006	0.001	°0.884

**Table 5:** Electron density ρ (in e/bohr^3^), Laplacian ∇^2^ρ (in e/bohr^5^), potential energy density V(r), Lagrangian kinetic energy G, and energy density H (in hartree/bohr^3^) at the bond critical points (3, −1), corresponding to the I⋯Pt interactions with d(I⋯I) (in Å) in **1**∙4I_2_, **2**∙2CHI_3_, **3**∙2CHI_3_, and **4**∙4I_2_.

Structure	Interaction	d(I⋯I)	ρ	∇^2^ρ	G	V	H	|V|/G
**1**∙4I_2_	* I1S⋯I1–Pt1*	* 3.9255 *	* 0.009 *	* 0.024 *	* 0.004 *	−*0.003*	* 0.001 *	* 0.663 *
	I2S⋯I1–Pt1	3.2362	0.029	0.041	0.014	−0.018	−0.004	1.259
	I3S⋯I1S–I2S	3.4484	0.020	0.044	0.012	−0.012	0.000	1.044
	I4S⋯I1–Pt1	3.4568	0.019	0.045	0.012	−0.012	0.000	1.031
**2**∙2CHI_3_	* I1S⋯I1–Pt1*	* 3.9138 *	* 0.008 *	* 0.029 *	* 0.006 *	−*0.004*	* 0.002 *	* 0.768 *
	I2S⋯I1–Pt1	3.5503	0.015	0.043	0.010	−0.010	0.000	0.952
	I3S⋯I1–Pt1	3.5687	0.014	0.043	0.010	−0.009	0.001	0.934
**3**∙2CHI_3_	* I1S⋯I1–Pt1*	* 3.7811 *	* 0.010 *	* 0.033 *	* 0.007 *	−*0.006*	* 0.001 *	* 0.815 *
	I2S⋯I1–Pt1	3.5559	0.015	0.044	0.010	−0.010	0.000	0.942
	I3S⋯I1–Pt1	3.7176	0.011	0.036	0.008	−0.007	0.001	0.852
	I4S⋯I1–Pt1	3.5675	0.015	0.042	0.010	−0.009	0.001	0.932
	I5S⋯I1A–Pt1A	3.5882	0.014	0.042	0.010	−0.009	0.001	0.924
	I6S⋯I1A–Pt1A	3.6094	0.014	0.041	0.009	−0.009	0.000	0.919
**4**∙4I_2_	* I1S⋯I1–Pt1*	* 3.7517 *	* 0.012 *	* 0.029 *	* 0.006 *	−*0.005*	* 0.001 *	* 0.795 *
	I2S⋯I1–Pt1	3.2980	0.026	0.041	0.013	−0.015	−0.002	1.196
	I3S⋯I1S–I2S	3.4385	0.020	0.045	0.012	–0.013	–0.001	1.051
	I4S⋯I1–Pt1	3.4783	0.017	0.046	0.011	–0.011	0.000	0.991

**Table 6:** Scaled symmetry adapted perturbation theory energies of zero-order E_sSAPT0_ and their electrostatic E_elst_, exchange E_exch_, induction E_ind_, and dispersion E_disp_ components, all in kcal/mol, corresponding to the I⋯(I–Pt) bifurcates in **1**∙(I_2_)_2_, **2**∙(CHI_3_)_2_, **3**∙(CHI_3_)_2_, and **4**∙(I_2_)_2_ models from **1**∙4I_2_, **2**∙2CHI_3_, **3**∙2CHI_3_, and **4**∙4I_2_, respectively.

Cluster	Interaction	E_sSAPT0_	E_elst_	E_exch_	E_ind_	E_disp_	E_disp_/E_elst_
**1**∙(I_2_)_2_	I2S–I1S⋯(I1–Pt1)	–9.55	–7.77	14.25	–4.32	–11.72	1.51
**2**∙(CHI_3_)_2_	C1S–I1S⋯(I1–Pt1)	–8.58	–5.97	12.58	–2.93	–12.27	2.06
**3**∙(CHI_3_)_2_	C1S–I1S⋯(I1–Pt1)	–8.80	–7.39	14.34	–3.40	–12.35	1.67
**4**∙(I_2_)_2_	I2S–I1S⋯(I1–Pt1)	–9.47	–7.26	14.07	–4.17	–12.11	1.67

## Data Availability

The data underlying the results presented in this paper are not publicly available at this time but may be obtained from the authors upon reasonable request.

## Supplementary Materials

The following supporting information can be downloaded at https://www.mdpi.com/article/10.3390/ijms26104555/s1.

## Abbreviations

The following abbreviations are used in this manuscript:

XRD	X-ray diffraction
XB	halogen bonding, halogen bond
DFT	density functional theory
GAPW	Gaussian augmented plane waves
COD	1,5-cycloocatadiene
FIB	1,4-diiodotetrafluorobenzene
IUPAC	International Union of Pure and Applied Chemistry
RT	room temperature
DKH2	Douglas–Kroll–Hess second-order scalar relativistic
QTAIM	quantum theory of atoms-in-molecules
BCP	bond critical point
RDG	reduced density gradient
NCI	noncovalent interaction (analysis)
ELF	electron localization function
sSAPT0	scaled symmetry adapted perturbation theory (zero order)
NMR	nuclear magnetic resonance
TLC	thin-layer chromatography
HRESI^+^-MS	high-resolution electrospray ionization–mass spectroscopy (positive ions)

## References

[1] Desiraju G.R., Ho P.S., Kloo L., Legon A.C., Marquardt R., Metrangolo P., Politzer P., Resnati G., Rissanen K. (2013). Definition of the halogen bond (IUPAC Recommendations 2013). Pure Appl. Chem..

[2] Cavallo G., Metrangolo P., Milani R., Pilati T., Priimagi A., Resnati G., Terraneo G. (2016). The Halogen Bond. Chem. Rev..

[3] Politzer P., Murray J. (2019). An Overview of Strengths and Directionalities of Noncovalent Interactions: σ-Holes and π-Holes. Crystals.

[4] Wang H., Bisoyi H.K., Urbas A.M., Bunning T.J., Li Q. (2019). The Halogen Bond: An Emerging Supramolecular Tool in the Design of Functional Mesomorphic Materials. Chem.-A Eur. J..

[5] Mukherjee A., Sanz-Matias A., Velpula G., Waghray D., Ivasenko O., Bilbao N., Harvey J.N., Mali K.S., De Feyter S. (2019). Halogenated building blocks for 2D crystal engineering on solid surfaces: Lessons from hydrogen bonding. Chem. Sci..

[6] Gilday L.C., Robinson S.W., Barendt T.A., Langton M.J., Mullaney B.R., Beer P.D. (2015). Halogen Bonding in Supramolecular Chemistry. Chem. Rev..

[7] Mahmudov K.T., Kopylovich M.N., Guedes da Silva M.F.C., Pombeiro A.J.L. (2017). Non-covalent interactions in the synthesis of coordination compounds: Recent advances. Coord. Chem. Rev..

[8] Dalpiaz A., Pavan B., Ferretti V. (2017). Can pharmaceutical co-crystals provide an opportunity to modify the biological properties of drugs?. Drug Discov. Today.

[9] Ho P.S. (2017). Halogen bonding in medicinal chemistry: From observation to prediction. Future Med. Chem..

[10] Berger G., Soubhye J., Meyer F. (2015). Halogen bonding in polymer science: From crystal engineering to functional supramolecular polymers and materials. Polym. Chem..

[11] Tepper R., Schubert U.S. (2018). Halogen Bonding in Solution: Anion Recognition, Templated Self-Assembly, and Organocatalysis. Angew. Chem. Int. Ed..

[12] Bulfield D., Huber S.M. (2016). Halogen Bonding in Organic Synthesis and Organocatalysis. Chem.-A Eur. J..

[13] Mahmudov K.T., Gurbanov A.V., Guseinov F.I., Guedes da Silva M.F.C. (2019). Noncovalent interactions in metal complex catalysis. Coord. Chem. Rev..

[14] Benz S., Poblador-Bahamonde A.I., Low-Ders N., Matile S. (2018). Catalysis with Pnictogen, Chalcogen, and Halogen Bonds. Angew. Chem. Int. Ed..

[15] Bennion J.C., Vogt L., Tuckerman M.E., Matzger A.J. (2016). Isostructural Cocrystals of 1,3,5-Trinitrobenzene Assembled by Halogen Bonding. Cryst. Growth Des..

[16] Landenberger K.B., Bolton O., Matzger A.J. (2015). Energetic-energetic cocrystals of diacetone diperoxide (DADP): Dramatic and divergent sensitivity modifications via cocrystallization. J. Am. Chem. Soc..

[17] Sivchik V.V., Solomatina A.I., Chen Y.T., Karttunen A.J., Tunik S.P., Chou P.T., Koshevoy I.O. (2015). Halogen Bonding to Amplify Luminescence: A Case Study Using a Platinum Cyclometalated Complex. Angew. Chem. Int. Ed..

[18] Sivchik V., Sarker R.K., Liu Z.Y., Chung K.Y., Grachova E.V., Karttunen A.J., Chou P.T., Koshevoy I.O. (2018). Improvement of the photophysical performance of platinum-cyclometalated complexes in halogen-bonded adducts. Chem.-A Eur. J..

[19] Kinzhalov M.A., Kashina M.V., Mikherdov A.S., Mozheeva E.A., Novikov A.S., Smirnov A.S., Ivanov D.M., Kryukova M.A., Ivanov A.Y., Smirnov S.N. (2018). Dramatically Enhanced Solubility of Halide-Containing Organometallic Species in Diiodomethane: The Role of Solvent⋯Complex Halogen Bonding. Angew. Chem. Int. Ed..

[20] Mikherdov A.S., Novikov A.S., Boyarskiy V.P., Kukushkin V.Y. (2020). The halogen bond with isocyano carbon reduces isocyanide odor. Nat. Commun..

[21] Metrangolo P., Resnati G. (2014). Type II halogen···halogen contacts are halogen bonds. IUCrJ.

[22] Hassel O. (1970). Structural aspects of interatomic charge-transfer bonding. Science.

[23] Ivanov D.M., Bokach N.A., Kukushkin V.Y., Frontera A. (2022). Metal Centers as Nucleophiles: Oxymoron of Halogen Bond-Involving Crystal Engineering. Chem.-A Eur. J..

[24] Eliseeva A.A., Ivanov D.M., Rozhkov A.V., Ananyev I.V., Frontera A., Kukushkin V.Y. (2021). Bifurcated Halogen Bonding Involving Two Rhodium(I) Centers as an Integrated σ-Hole Acceptor. JACS Au.

[25] Ivanov D.M., Novikov A.S., Ananyev I.V., Kirina Y.V., Kukushkin V.Y. (2016). Halogen Bonding Between Metal Center and Halocarbon. Chem. Commun..

[26] Dabranskaya U., Ivanov D.M., Novikov A.S., Matveychuk Y.V., Bokach N.A., Kukushkin V.Y. (2019). Metal-Involving Bifurcated Halogen Bonding C–Br···η2(Cl–Pt). Cryst. Growth Des..

[27] Aliyarova I.S., Tupikina E.Y., Ivanov D.M., Kukushkin V.Y. (2022). Metal-Involving Halogen Bonding Including Gold(I) as a Nucleophilic Partner. The Case of Isomorphic Dichloroaurate(I)·Halomethane Cocrystals. Inorg. Chem..

[28] Katlenok E.A., Haukka M., Levin O.V., Frontera A., Kukushkin V.Y. (2020). Supramolecular Assembly of Metal Complexes by (Aryl)I⋯dz2[PtII] Halogen Bond. Chem.-A Eur. J..

[29] Katlenok E.A., Rozhkov A.V., Levin O.V., Haukka M., Kuznetsov M.L., Kukushkin V.Y. (2021). Halogen Bonding Involving Palladium(II) as an XB Acceptor. Cryst. Growth Des..

[30] Bulatova M., Ivanov D.M., Haukka M. (2021). Classics Meet Classics: Theoretical and Experimental Studies of Halogen Bonding in Adducts of Platinum(II) 1,5-Cyclooctadiene Halide Complexes with Diiodine, Iodoform, and 1,4-Diiodotetrafluorobenzene. Cryst. Growth Des..

[31] Bulatova M., Ivanov D.M., Rautiainen J.M., Kinzhalov M.A., Truong K.N., Lahtinen M., Haukka M. (2021). Studies of Nature of Uncommon Bifurcated I-I···(I-M) Metal-Involving Noncovalent Interaction in Palladium(II) and Platinum(II) Isocyanide Cocrystals. Inorg. Chem..

[32] Bondi A. (1964). van der Waals Volumes and Radii. J. Phys. Chem..

[33] Comas-Vilà G., Salvador P. (2024). Quantification of the Donor-Acceptor Character of Ligands by the Effective Fragment Orbitals. ChemPhysChem.

[34] Kashina M.V., Kinzhalov M.A., Smirnov A.S., Ivanov D.M., Novikov A.S., Kukushkin V.Y. (2019). Dihalomethanes as Bent Bifunctional XB/XB-Donating Building Blocks for Construction of Metal-involving Halogen Bonded Hexagons. Chem.-Asian J..

[35] Cheranyova A.M., Zelenkov L.E., Baykov S.V., Izotova Y.A., Ivanov D.M., Bokach N.A., Kukushkin V.Y. (2024). Intermolecular Metal-Involving Pnictogen Bonding: The Case of σ-(SbIII)-Hole···dz[PtII] Interaction. Inorg. Chem..

[36] Eliseeva A.A., Khazanova M.A., Cheranyova A.M., Aliyarova I.S., Kravchuk R.I., Oganesyan E.S., Ryabykh A.V., Maslova O.A., Ivanov D.M., Beznosyuk S.A. (2023). Metal-Involving Halogen Bonding Confirmed Using DFT Calculations with Periodic Boundary Conditions. Crystals.

[37] Bertolotti F., Shishkina A., Forni A., Gervasio G., Stash A., Tsirelson V. (2014). Intermolecular Bonding Features in Solid Iodine. Cryst. Growth Des..

[38] Johnson M.T., Džolić Z., Cetina M., Wendt O.F., Öhrström L., Rissanen K. (2012). Neutral Organometallic Halogen Bond Acceptors: Halogen Bonding in Complexes of PCPPdX (X = Cl, Br, I) with Iodine (I2), 1,4-Diiodotetrafluorobenzene (F4DIBz), and 1,4-Diiodooctafluorobutane (F8DIBu). Cryst. Growth Des..

[39] Spackman M.A., Jayatilaka D. (2009). Hirshfeld surface analysis. CrystEngComm.

[40] McKinnon J.J., Jayatilaka D., Spackman M.A. (2007). Towards quantitative analysis of intermolecular interactions with Hirshfeld surfaces. Chem. Commun..

[41] Spackman P.R., Turner M.J., McKinnon J.J., Wolff S.K., Grimwood D.J., Jayatilaka D., Spackman M.A. (2021). CrystalExplorer: A program for Hirshfeld surface analysis, visualization and quantitative analysis of molecular crystals. J. Appl. Crystallogr..

[42] Perdew J.P., Burke K., Ernzerhof M. (1996). Generalized gradient approximation made simple. Phys. Rev. Lett..

[43] Grimme S., Antony J., Ehrlich S., Krieg H. (2010). A consistent and accurate ab initio parametrization of density functional dispersion correction (DFT-D) for the 94 elements H-Pu. J. Chem. Phys..

[44] Grimme S., Ehrlich S., Goerigk L. (2011). Effect of the damping function in dispersion corrected density functional theory. J. Comput. Chem..

[45] Jorge F.E., Canal Neto A., Camiletti G.G., MacHado S.F. (2009). Contracted Gaussian basis sets for Douglas-Kroll-Hess calculations: Estimating scalar relativistic effects of some atomic and molecular properties. J. Chem. Phys..

[46] Barros C.L., De Oliveira P.J.P., Jorge F.E., Canal Neto A., Campos M. (2010). Gaussian basis set of double zeta quality for atoms Rb through Xe: Application in non-relativistic and relativistic calculations of atomic and molecular properties. Mol. Phys..

[47] de Berrêdo R.C., Jorge F.E. (2010). All-electron double zeta basis sets for platinum: Estimating scalar relativistic effects on platinum(II) anticancer drugs. J. Mol. Struct. THEOCHEM.

[48] Pritchard B.P., Altarawy D., Didier B., Gibson T.D., Windus T.L. (2019). New Basis Set Exchange: An Open, Up-to-Date Resource for the Molecular Sciences Community. J. Chem. Inf. Model..

[49] Hutter J., Iannuzzi M., Schiffmann F., VandeVondele J. (2014). _cp_2_k_ atomistic simulations of condensed matter systems. Wiley Interdiscip. Rev. Comput. Mol. Sci..

[50] Kühne T.D., Iannuzzi M., Del Ben M., Rybkin V.V., Seewald P., Stein F., Laino T., Khaliullin R.Z., Schütt O., Schiffmann F. (2020). CP2K: An electronic structure and molecular dynamics software package -Quickstep: Efficient and accurate electronic structure calculations. J. Chem. Phys..

[51] Frigo M., Johnson S.G. (2005). The Design and Implementation of FFTW3. Proc. IEEE.

[52] Vandevondele J., Krack M., Mohamed F., Parrinello M., Chassaing T., Hutter J. (2005). Quickstep: Fast and accurate density functional calculations using a mixed Gaussian and plane waves approach. Comput. Phys. Commun..

[53] Borštnik U., Vandevondele J., Weber V., Hutter J. (2014). Sparse matrix multiplication: The distributed block-compressed sparse row library. Parallel Comput..

[54] Schütt O., Messmer P., Hutter J., VandeVondele J. (2016). GPU-Accelerated Sparse Matrix-Matrix Multiplication for Linear Scaling Density Functional Theory. Electronic Structure Calculations on Graphics Processing Units.

[55] Goerigk L., Hansen A., Bauer C., Ehrlich S., Najibi A., Grimme S. (2017). A look at the density functional theory zoo with the advanced GMTKN55 database for general main group thermochemistry, kinetics and noncovalent interactions. Phys. Chem. Chem. Phys..

[56] Lippert G., Hutter J., Parrinello M. (1999). The Gaussian and augmented-plane-wave density functional method for ab initio molecular dynamics simulations. Theor. Chem. Acc..

[57] Espinosa E., Alkorta I., Elguero J., Molins E. (2002). From weak to strong interactions: A comprehensive analysis of the topological and energetic properties of the electron density distribution involving X-H⋯F-Y systems. J. Chem. Phys..

[58] Johnson E.R., Keinan S., Mori-Sánchez P., Contreras-García J., Cohen A.J., Yang W. (2010). Revealing noncovalent interactions. J. Am. Chem. Soc..

[59] Becke A.D., Edgecombe K.E. (1990). A simple measure of electron localization in atomic and molecular systems. J. Chem. Phys..

[60] Silvi B., Savin A. (1994). Classification of chemical bonds based on topological analysis of electron localization functions. Nature.

[61] Savin A., Nesper R., Wengert S., Fässler T.F. (1997). ELF: The Electron Localization Function. Angew. Chemie Int. Ed. Engl..

[62] Jeziorski B., Moszynski R., Szalewicz K. (1994). Perturbation Theory Approach to Intermolecular Potential Energy Surfaces of van der Waals Complexes. Chem. Rev..

[63] Parker T.M., Burns L.A., Parrish R.M., Ryno A.G., Sherrill C.D. (2014). Levels of symmetry adapted perturbation theory (SAPT). I. Efficiency and performance for interaction energies. J. Chem. Phys..

[64] Weigend F., Ahlrichs R. (2005). Balanced basis sets of split valence, triple zeta valence and quadruple zeta valence quality for H to Rn: Design and assessment of accuracy. Phys. Chem. Chem. Phys..

[65] Weigend F. (2006). Accurate Coulomb-fitting basis sets for H to Rn. Phys. Chem. Chem. Phys..

[66] Sarju J., Arbour J., Sayer J., Rohrmoser B., Scherer W., Wagner G. (2008). Synthesis and characterisation of mixed ligand Pt(ii) and Pt(iv) oxadiazoline complexes. Dalt. Trans..

[67] Hofmann K., Bugge G. (1907). Vergleich der Nitrile und Isonitrile im Verhalten gegen Metallsalze, ein Beitrag zur Konstitution der Doppelcyanide. Berichte Der Dtsch. Chem. Ges..

[68] Dolomanov O.V., Bourhis L.J., Gildea R.J., Howard J.A.K., Puschmann H. (2009). OLEX2: A complete structure solution, refinement and analysis program. J. Appl. Crystallogr..

[69] Sheldrick G.M. (2015). SHELXT-Integrated space-group and crystal-structure determination. Acta Crystallogr. Sect. A Found. Crystallogr..

[70] Sheldrick G.M. (2008). A short history of SHELX. Acta Crystallogr. Sect. A Found. Crystallogr..

[71] Allen F.H., Bruno I.J. (2010). Bond lengths in organic and metal-organic compounds revisited: X-H bond lengths from neutron diffraction data. Acta Crystallogr. Sect. B Struct. Sci..

[72] Barysz M., Sadlej A.J. (2001). Two-component methods of relativistic quantum chemistry: From the Douglas-Kroll approximation to the exact two-component formalism. J. Mol. Struct. THEOCHEM.

[73] Reiher M. (2012). Relativistic Douglas-Kroll-Hess theory. Wiley Interdiscip. Rev. Comput. Mol. Sci..

[74] Bader R.F.W., Nguyen-Dang T.T. (1981). Quantum Theory of Atoms in Molecules–Dalton Revisited. Adv. Quantum Chem..

[75] Bader R.F.W. (1991). A Quantum Theory of Molecular Structure and Its Applications. Chem. Rev..

[76] Lu T., Chen F. (2012). Multiwfn: A Multifunctional Wavefunction Analyzer. J. Comput. Chem..

[77] Smith D.G.A., Burns L.A., Simmonett A.C., Parrish R.M., Schieber M.C., Galvelis R., Kraus P., Kruse H., Di Remigio R., Alenaizan A. (2020). Psi4 1.4: Open-source software for high-throughput quantum chemistry. J. Chem. Phys..

[78] Suslonov V.V., Soldatova N.S., Ivanov D.M., Galmés B., Frontera A., Resnati G., Postnikov P.S., Kukushkin V.Y., Bokach N.A. (2021). Diaryliodonium Tetrachloroplatinates(II): Recognition of a Trifurcated Metal-Involving μ3-I···(Cl,Cl,Pt) Halogen Bond. Cryst. Growth Des..

[79] Bokach N.A., Kukushkin V.Y. (2013). Coordination chemistry of dialkylcyanamides: Binding properties, synthesis of metal complexes, and ligand reactivity. Coord. Chem. Rev..

