# Supplementary material for: Metal-Involving Bifurcated Halogen Bonding with Iodide and Platinum(II) Center
Source: Int J Mol Sci. 2025 May 9;26(10):4555. doi: 10.3390/ijms26104555 (PMC12111071; doi:10.3390/ijms26104555)
Supplement: Supplementary file 1 [file ijms-26-04555-s001.zip › Supplementary Materials.pdf]

Supplementary Materials

Metal-involving bifurcated halogen bonding with iodide and platinum(II) center

Maria A. Kryukova [1], Margarita B. Kostareva [1], Anna M. Cheraneva,[1] Marina A. Khazanova [1], Anton V. Rozhkov [1], Daniil M. Ivanov [1,]*

[1] Institute of Chemistry, Saint Petersburg State University 7/9 Universitetskaya Nab., Saint Petersburg 199034 (Russian Federation); d.m.ivanov@spbu.ru
* Correspondence: d.m.ivanov@spbu.ru

Table of contents:

S1. Crystal data and structure refinement 2

S2. ^{1}H NMR, $^{13}C\{^{1}H\}$ NMR and ^{195}Pt NMR spectra of compounds 3

S3. IR spectra of compounds 8

S4. Mass-spectra of compounds 10

S5. The environment of complex **3** (Pt1A) 11

S6. Cartesian coordinates of theoretical models 12

S1. Crystal data and structure refinement

Table S1. Crystal data and structure refinement for **1**·$4I_2$, **2**·$2CHI_3$, **3**·$2CHI_3$, **4**·$4I_2$.

Identification code	**1**·$4I_2$	**2**·$2CHI_3$	**3**·$2CHI_3$	**4**·$4I_2$
CCDC number	2441327	2441328	2441329	2441330
Empirical formula	$C_6H_{12}I_{10}N_4Pt$	$C_{12}H_{22}I_8N_4Pt$	$C_{16}H_{12}I_8N_2Pt$	$C_{14}H_8Cl_2I_{10}N_2Pt$
Formula weight	1604.29	1432.62	1442.57	1739.21
Temperature, K	100(2)	100(2)	100(2)	100(2)
Crystal system	monoclinic	triclinic	monoclinic	triclinic
Space group	$P2_1/n$	P-1	$P2_1/c$	P-1
a, Å	8.1527(10)	8.8689(5)	14.9048(10)	8.3871(2)
b, Å	17.635(2)	10.1937(5)	10.0110(5)	9.5944(3)
c, Å	9.7882(11)	10.4526(5)	19.0509(14)	10.8200(3)
α, °	90	62.101(5)	90	113.505(3)
β, °	93.660(10)	87.064(4)	105.150(7)	98.091(2)
γ, °	90	64.956(5)	90	96.933(2)
Volume, Å3	1404.4(3)	743.80(8)	2743.8(3)	775.34(4)
Z	2	1	4	1
ρcalc, g/cm3	3.794	3.198	3.492	3.725
μ, mm–1	15.986	13.024	14.122	14.659
F(000)	1368	624	2496	748
Crystal size, mm3	0.2 × 0.09 × 0.08	0.15 × 0.13 × 0.1	0.15 × 0.14 × 0.1	0.2 × 0.18 × 0.15
Radiation	Mo Kα (λ = 0.71073)	Mo Kα (λ = 0.71073)	Mo Kα (λ = 0.71073)	Mo Kα (λ = 0.71073)
2Θ range for data	5.514 to 50.992	5.158 to 54.996	5.664 to 51.994	5.824 to 61.754
collection, °	$-9 \le h \le 9$, $-21 \le k \le 17$, $-11 \le l \le 11$	$-11 \le h \le 10$, $-13 \le k \le 12$, $-11 \le l \le 13$	$-18 \le h \le 18$, $-12 \le k \le 12$, $-23 \le l \le 23$	$-11 \le h \le 12$, $-13 \le k \le 13$, $-15 \le l \le 15$
Index ranges	5636	6379	24143	14918
Reflections collected	2588 [R_{int} = 0.0245, R_{sigma} = 0.0319]	3416 [R_{int} = 0.0277, R_{sigma} = 0.0481]	5393 [R_{int} = 0.0577, R_{sigma} = 0.0462]	4469 [R_{int} = 0.0328, R_{sigma} = 0.0351]
Independent reflections	2588/0/99	3416/4/124	5393/0/241	4469/0/133
Data/restraints/parameters	1.077	1.027	1.085	1.04
Goodness-of-fit on F2	R_1 = 0.0265, wR_2 = 0.0599	R_1 = 0.0302, wR_2 = 0.0515	R_1 = 0.0326, wR_2 = 0.0660	R_1 = 0.0222, wR_2 = 0.0402
Final R indexes [I≥2σ (I)]	R_1 = 0.0296, wR_2 = 0.0613	R_1 = 0.0412, wR_2 = 0.0563	R_1 = 0.0428, wR_2 = 0.0722	R_1 = 0.0288, wR_2 = 0.0422
Final R indexes [all data]	2.42/–1.41	1.36/–1.23	2.19/–1.37	1.02/–0.90

S2. ^{1}H NMR, ^{13}C{^{1}H} NMR and ^{195}Pt NMR spectra of compounds

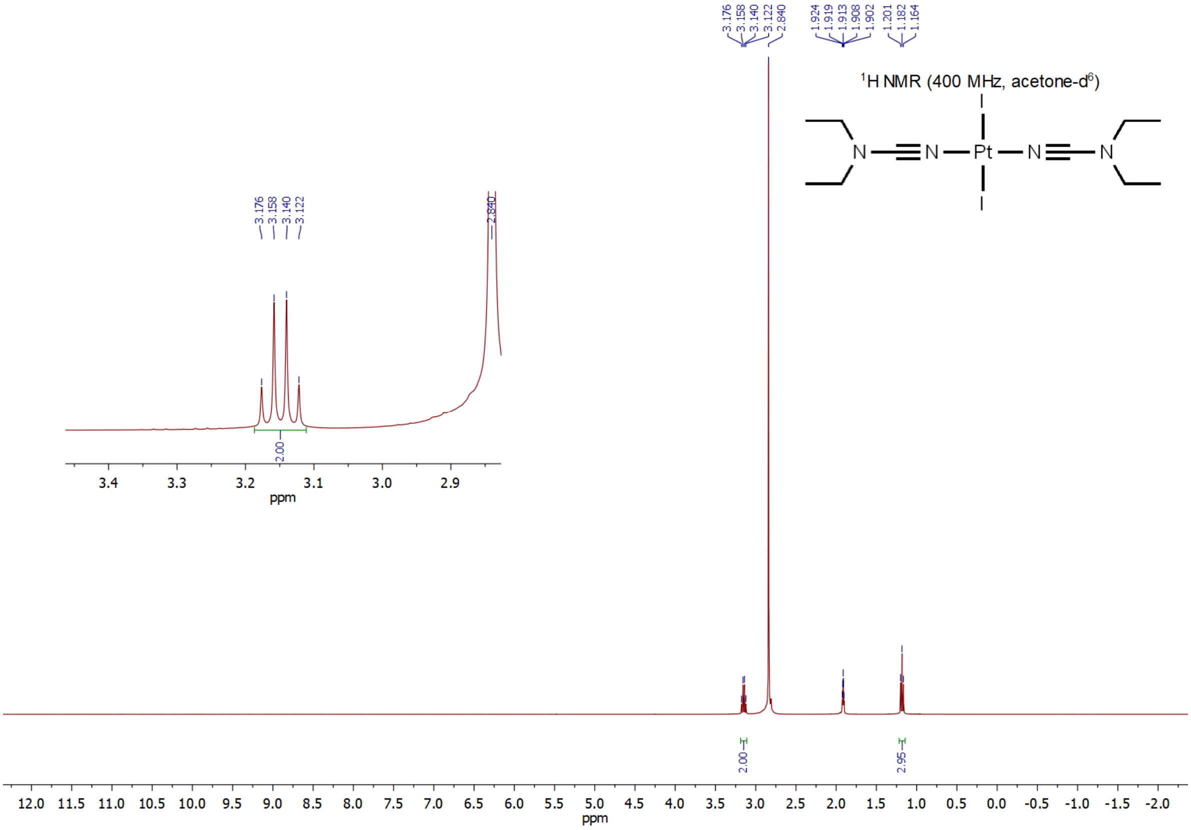

Figure S1. ^{1}H NMR (400 MHz, acetone-d^6) spectrum of **2**.

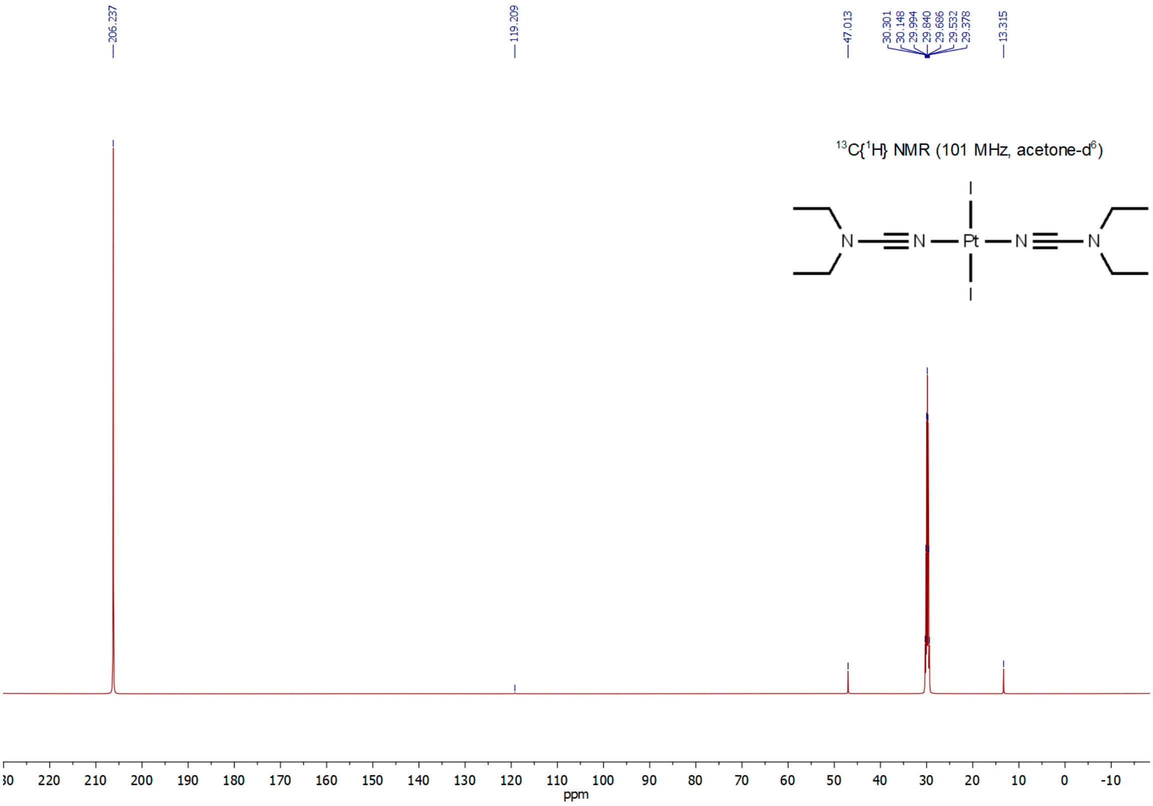

Figure S2. ^{13}C{^{1}H} NMR (101 MHz, acetone-d^6) spectrum of **2**.

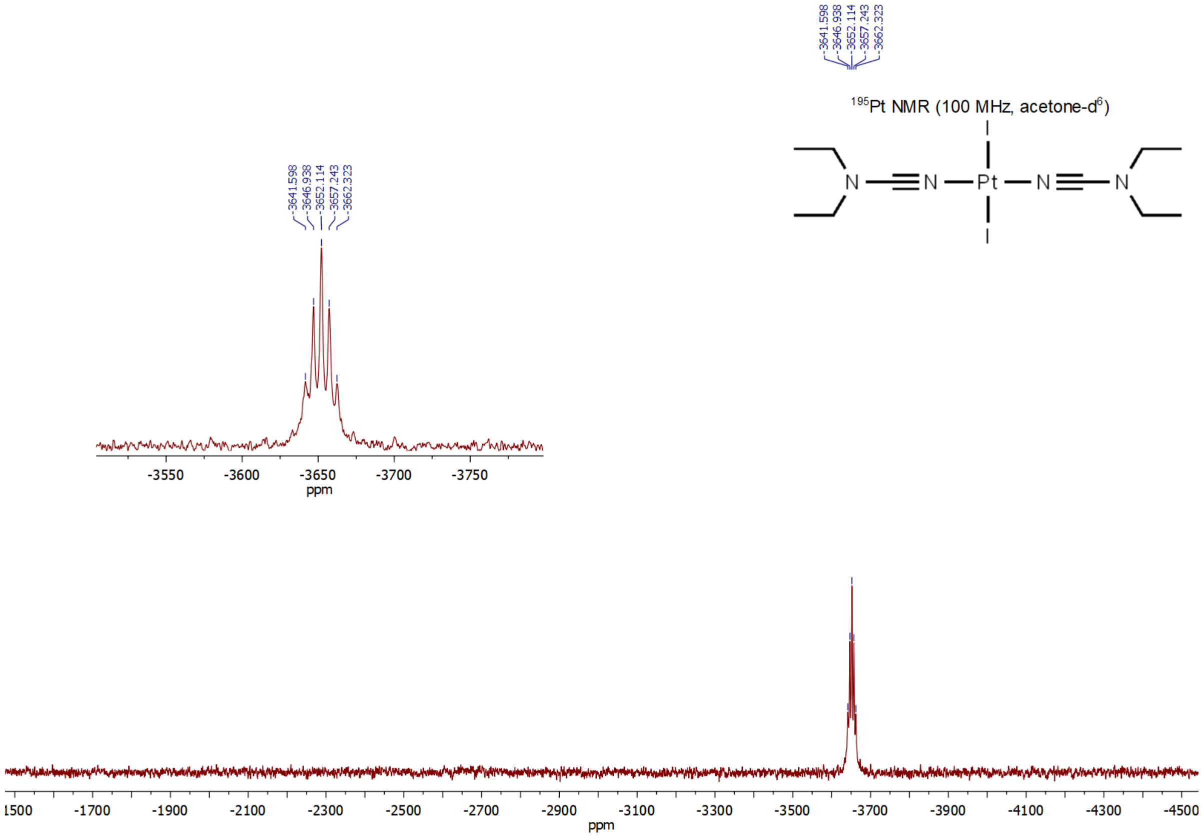

Figure S3. ^{195}Pt NMR (100 MHz, acetone-d^6) spectrum of **2**.

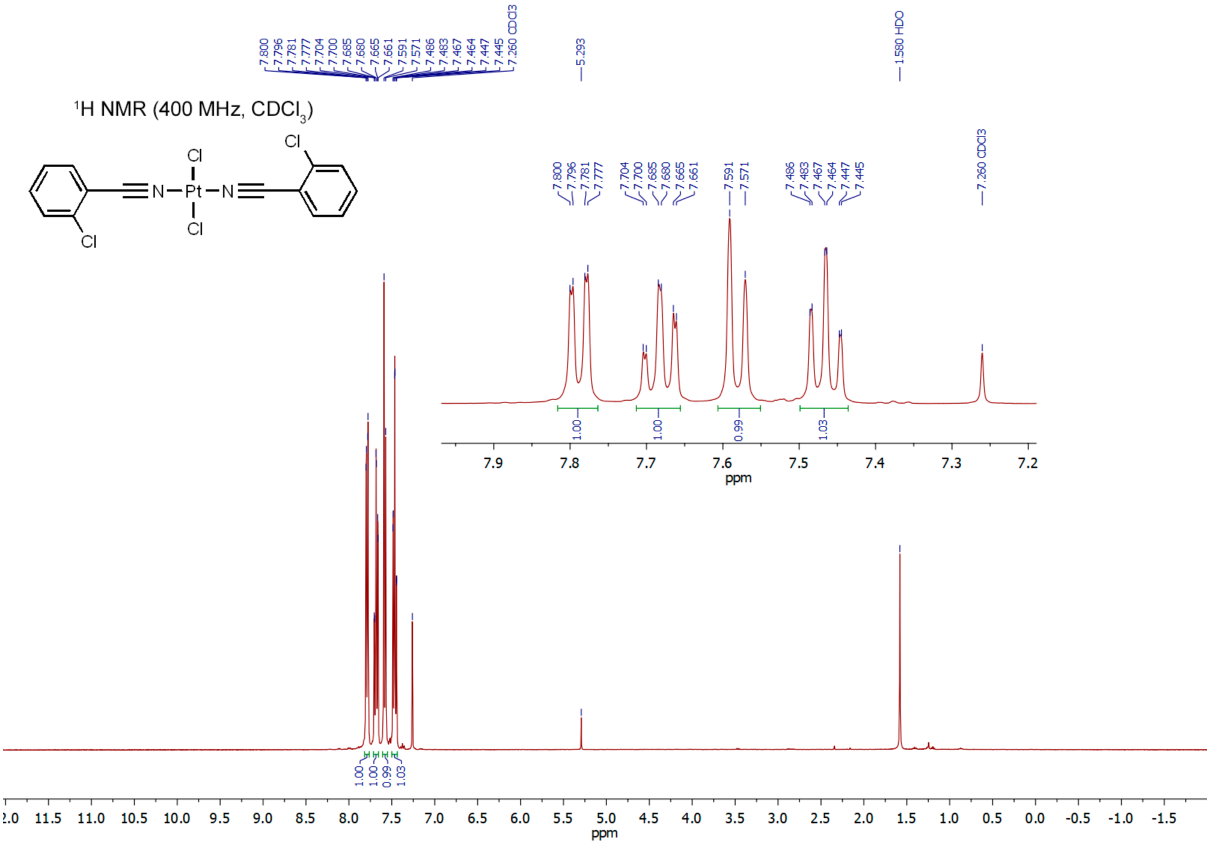

Figure S4. ^{1}H NMR (400 MHz, $CDCl_3$) spectrum of **2**.

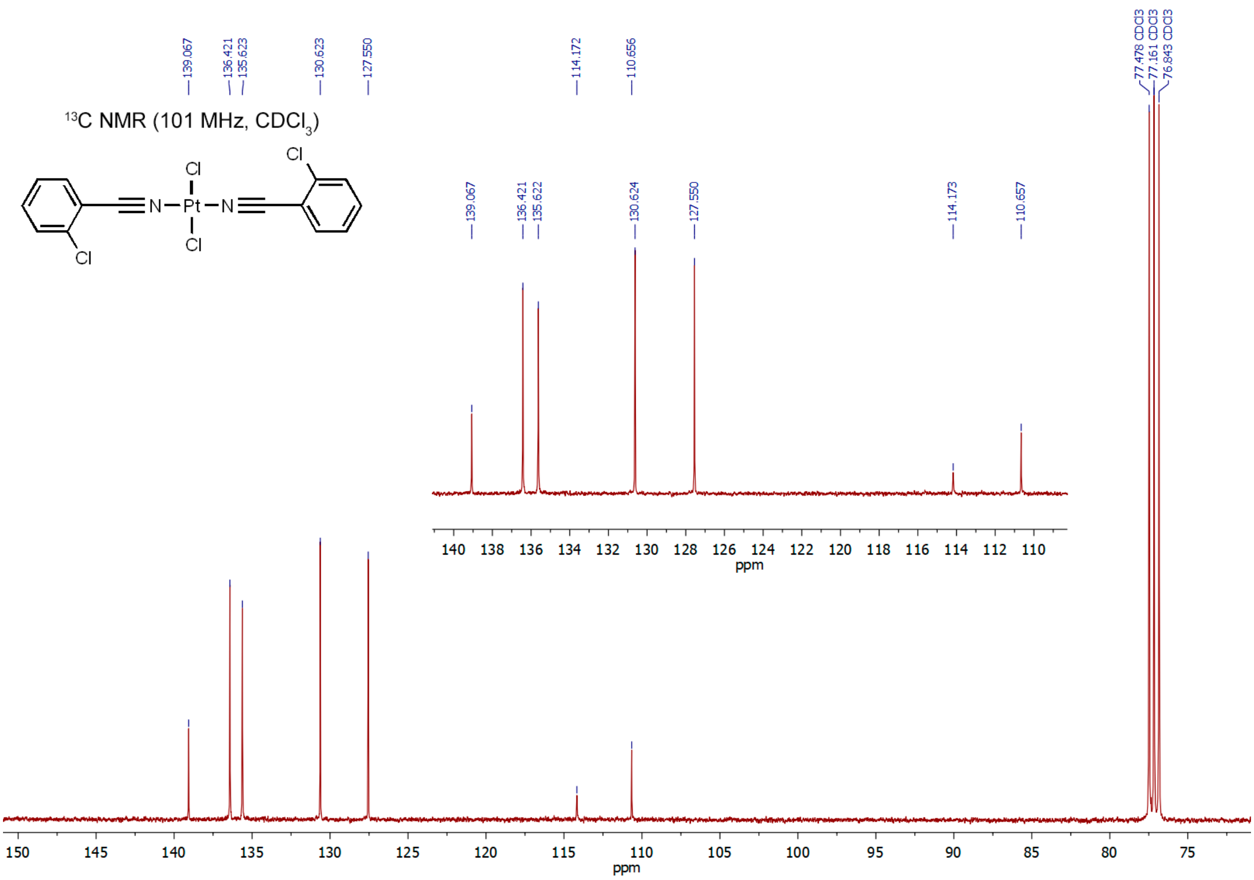

Figure S5. $^{13}C\{^1H\}$ NMR (101 MHz, $CDCl_3$) spectrum of *trans*-[$PtCl_2(2\text{-}ClC_6H_4CN)_2$].

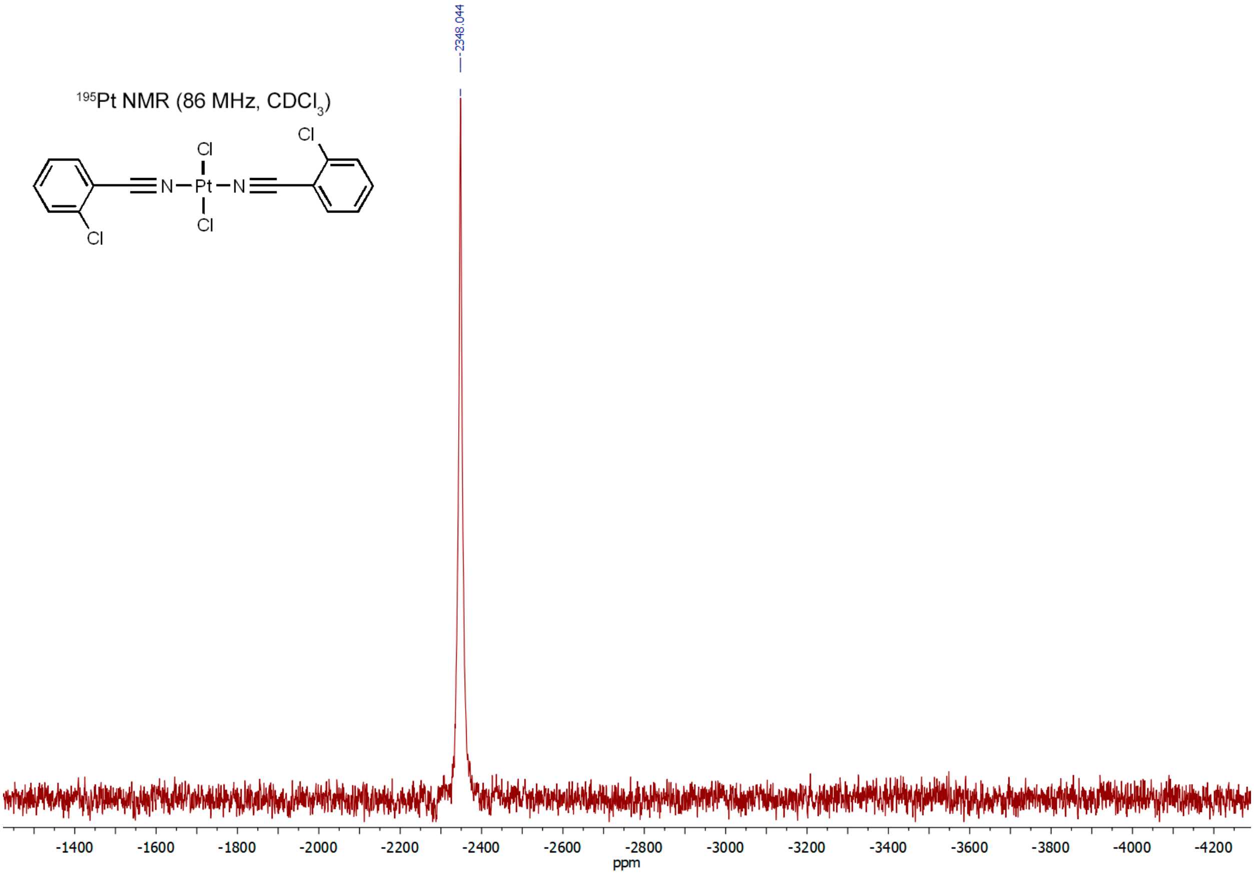

Figure S6. ^{195}Pt NMR (86 MHz, $CDCl_3$) spectrum of *trans*-[$PtCl_2(2\text{-}ClC_6H_4CN)_2$].

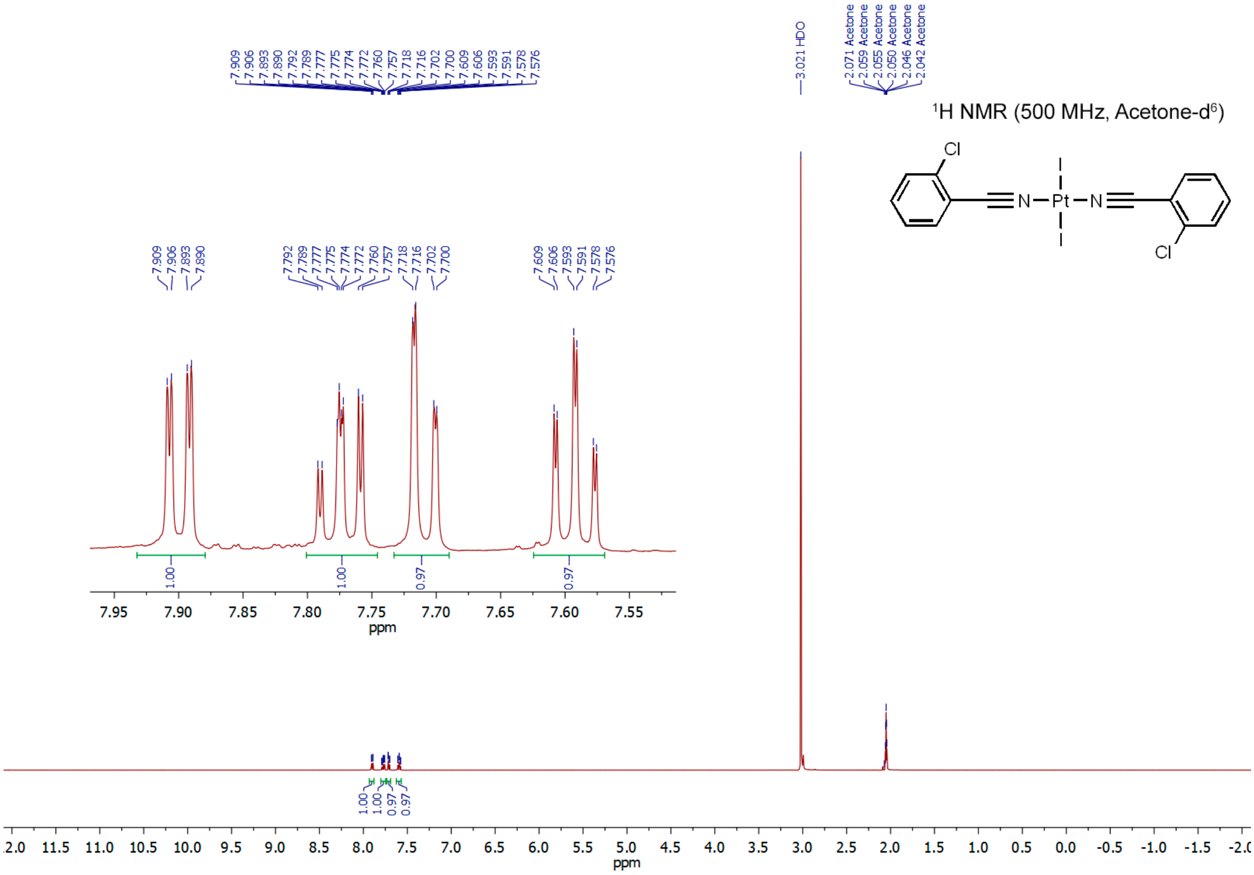

Figure S7. ^{1}H NMR (500 MHz, acetone-d^6) spectrum of **4**.

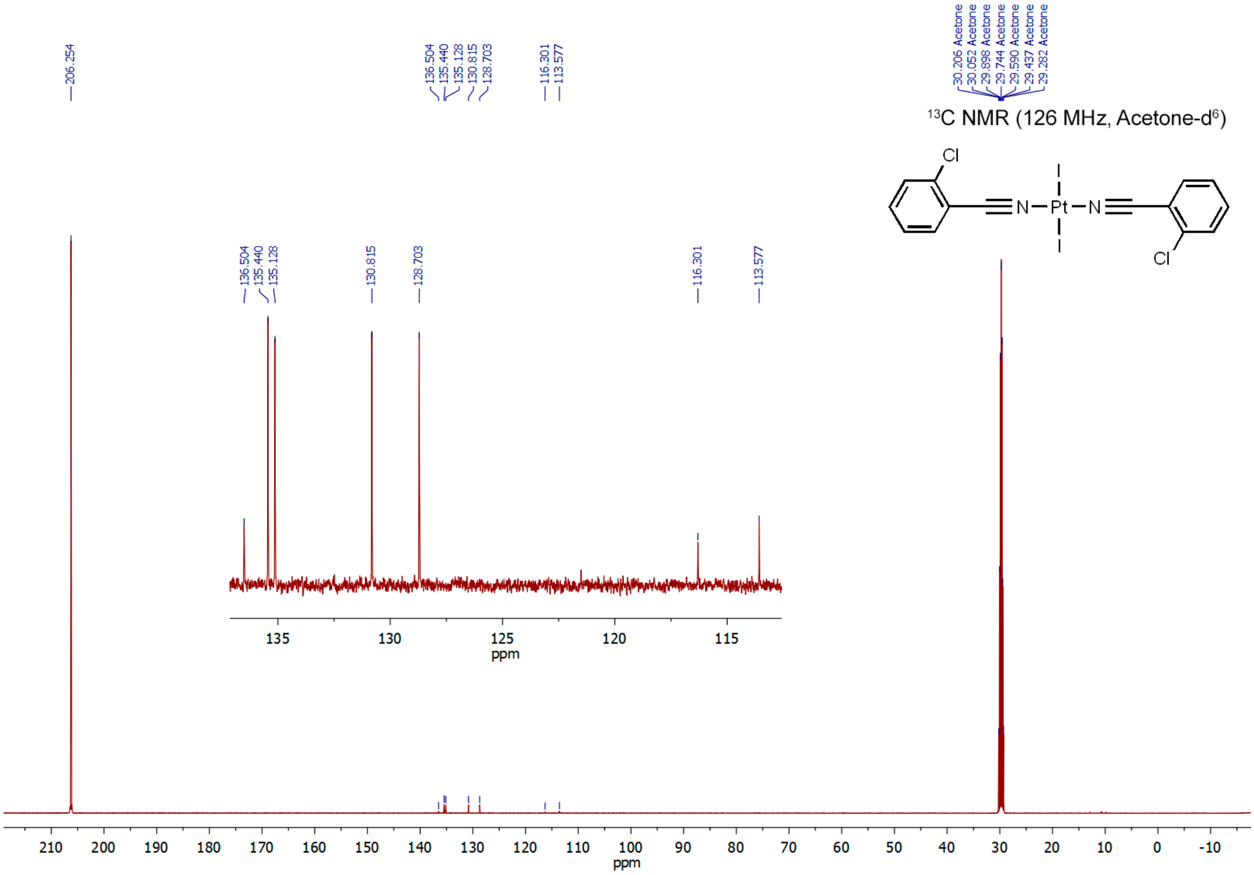

Figure S8. ^{13}C{^{1}H} NMR (126 MHz, acetone-d^6) spectrum of **4**.

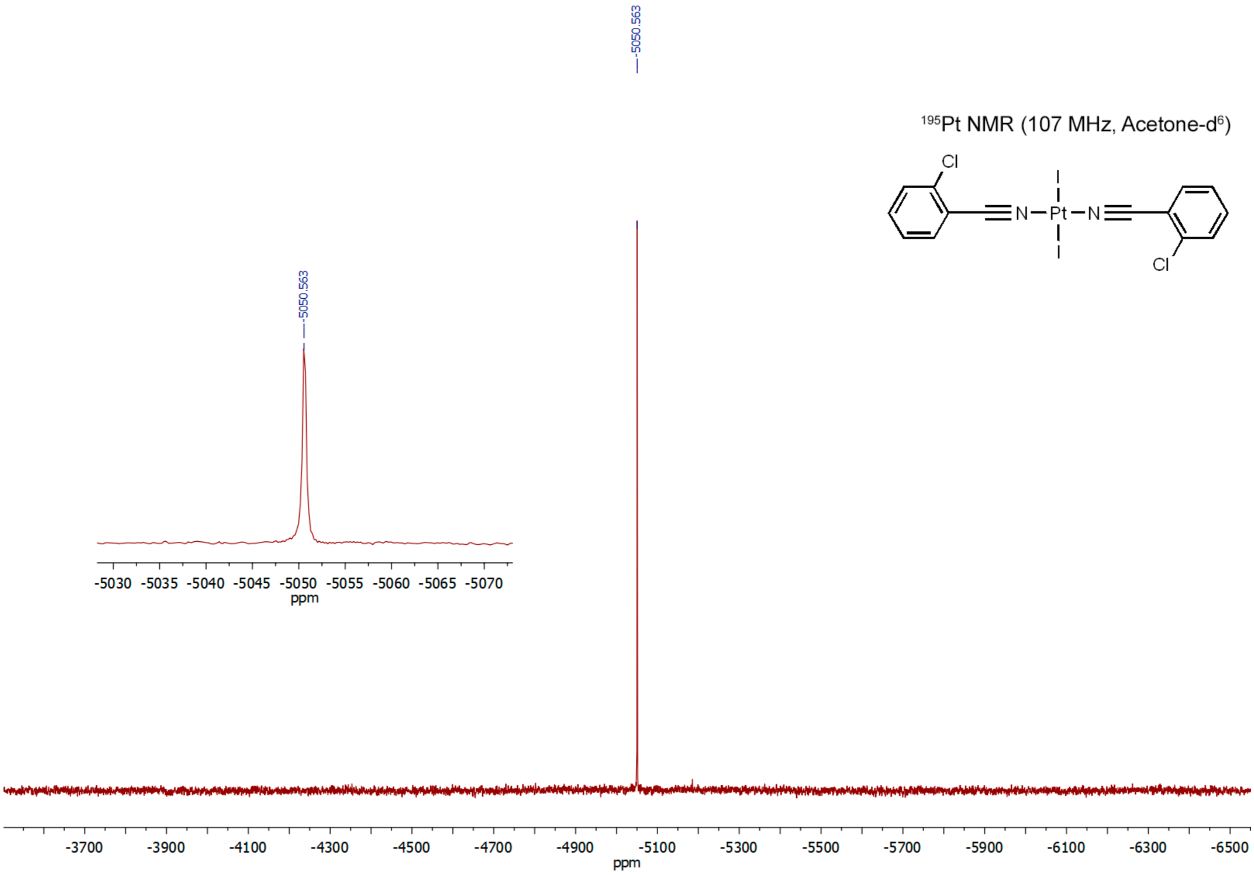

Figure S9. ^{195}Pt NMR (107 MHz, acetone-d^6) spectrum of **4**.

S3. IR spectra of compounds

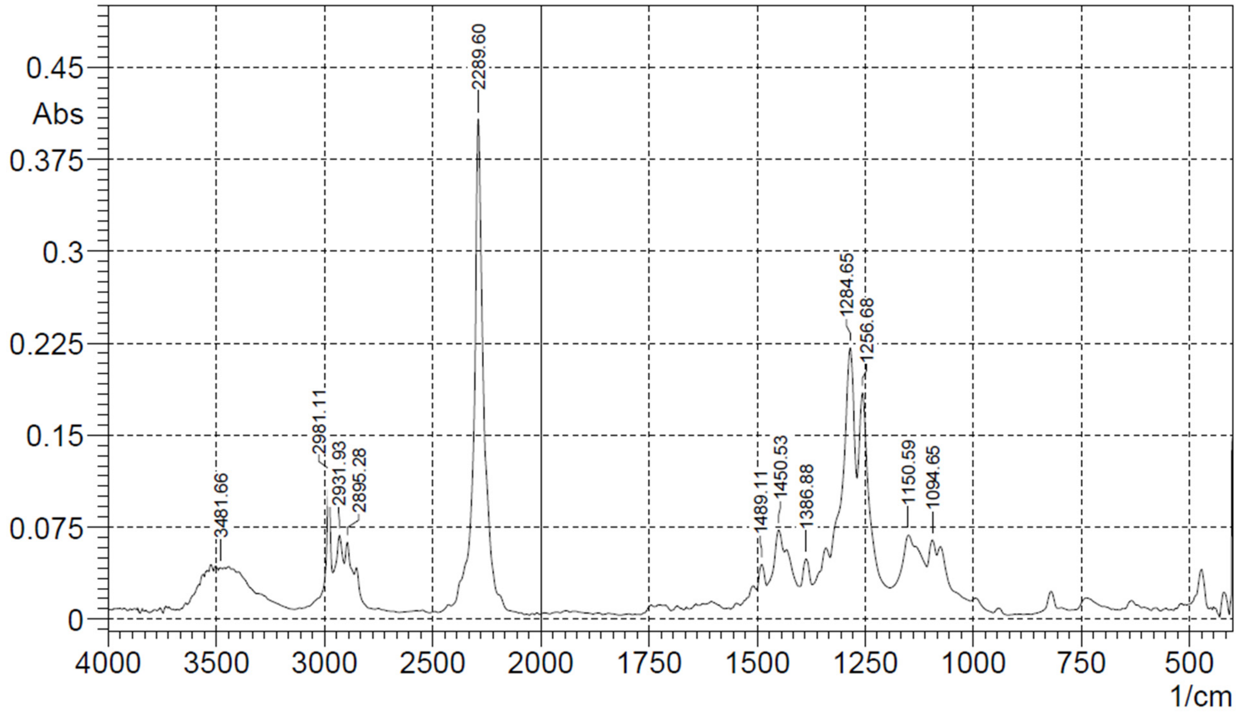

Figure S10. IR spectrum of **2** in CsI.

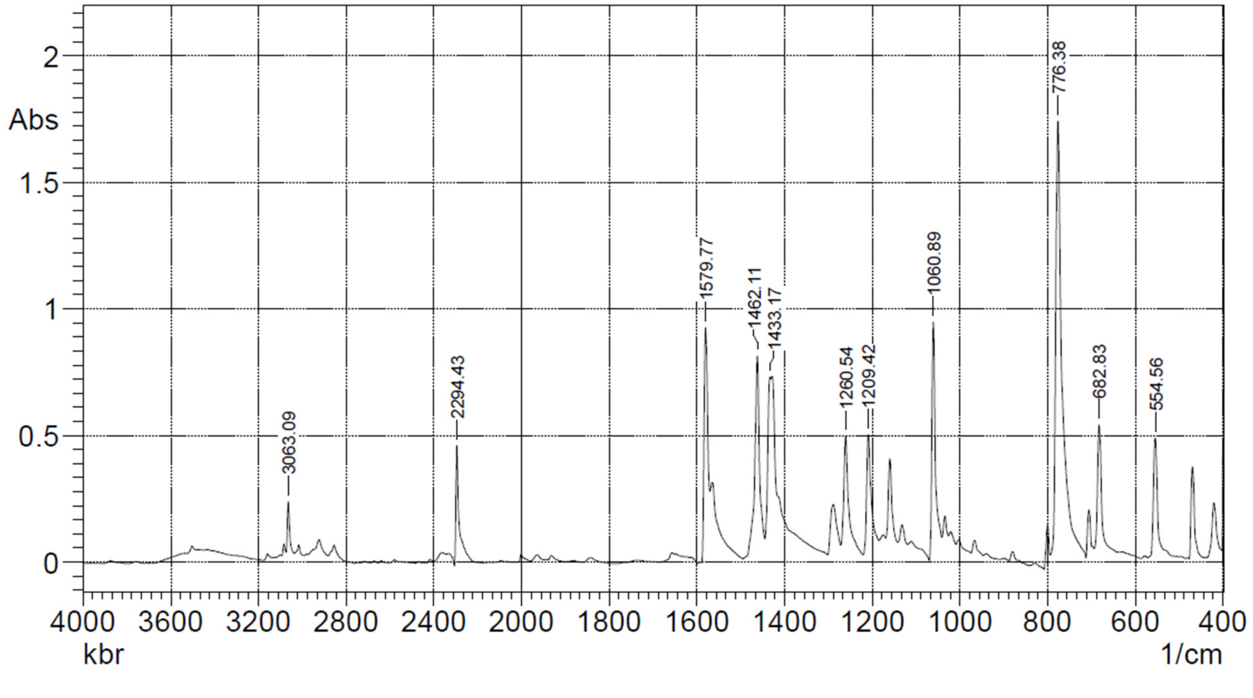

Figure S11. IR spectrum of *trans*-$[PtCl_2(2\text{-}ClC_6H_4CN)_2]$ in KBr.

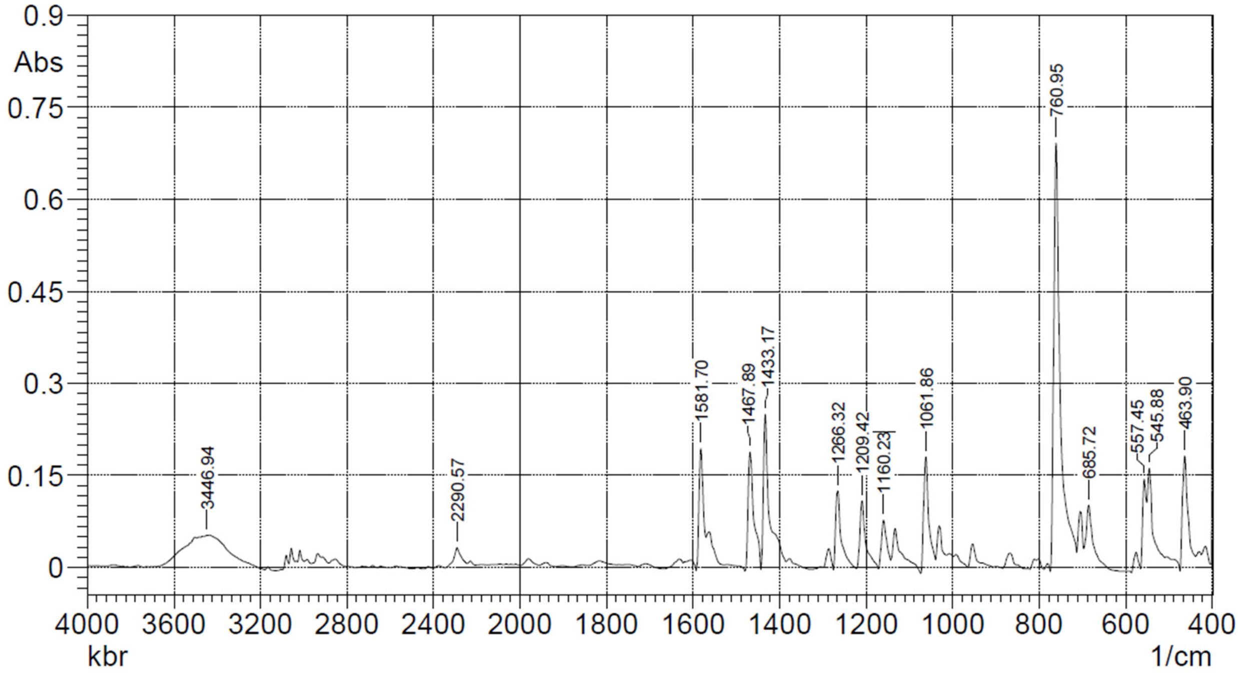

Figure S12. IR spectrum of **4** in KBr.

S4. Mass-spectra of compounds

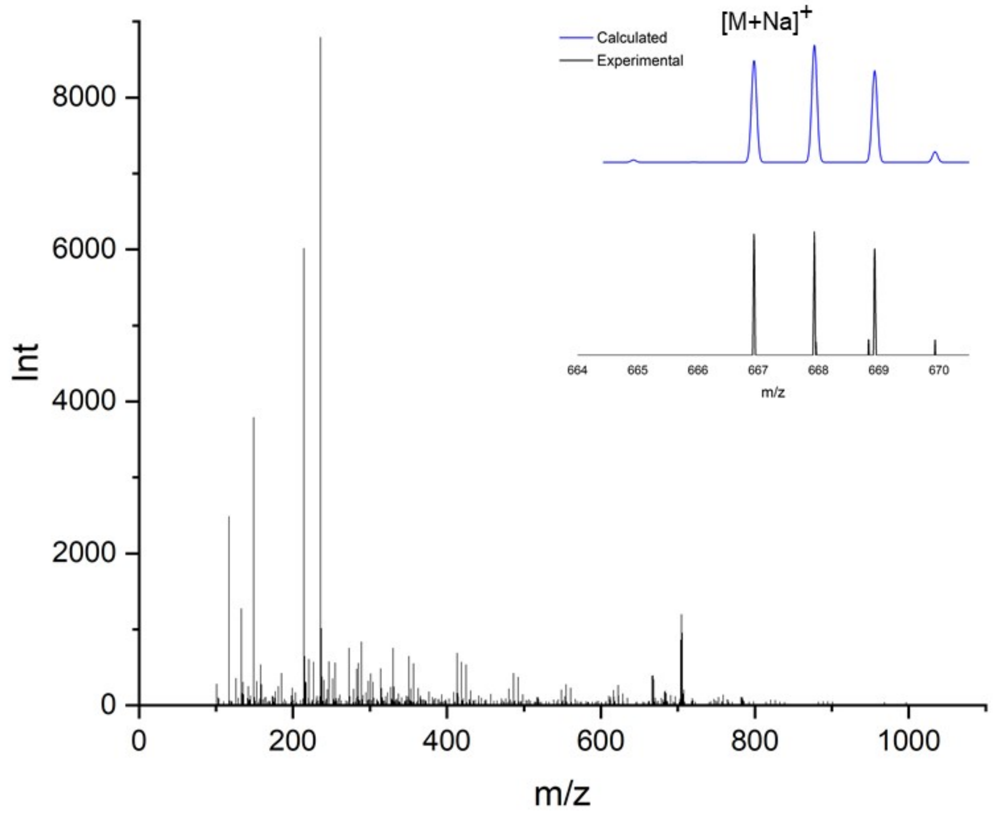

Figure S13. Mass-spectrum (ESI$^+$-HR) spectrum of **2**.

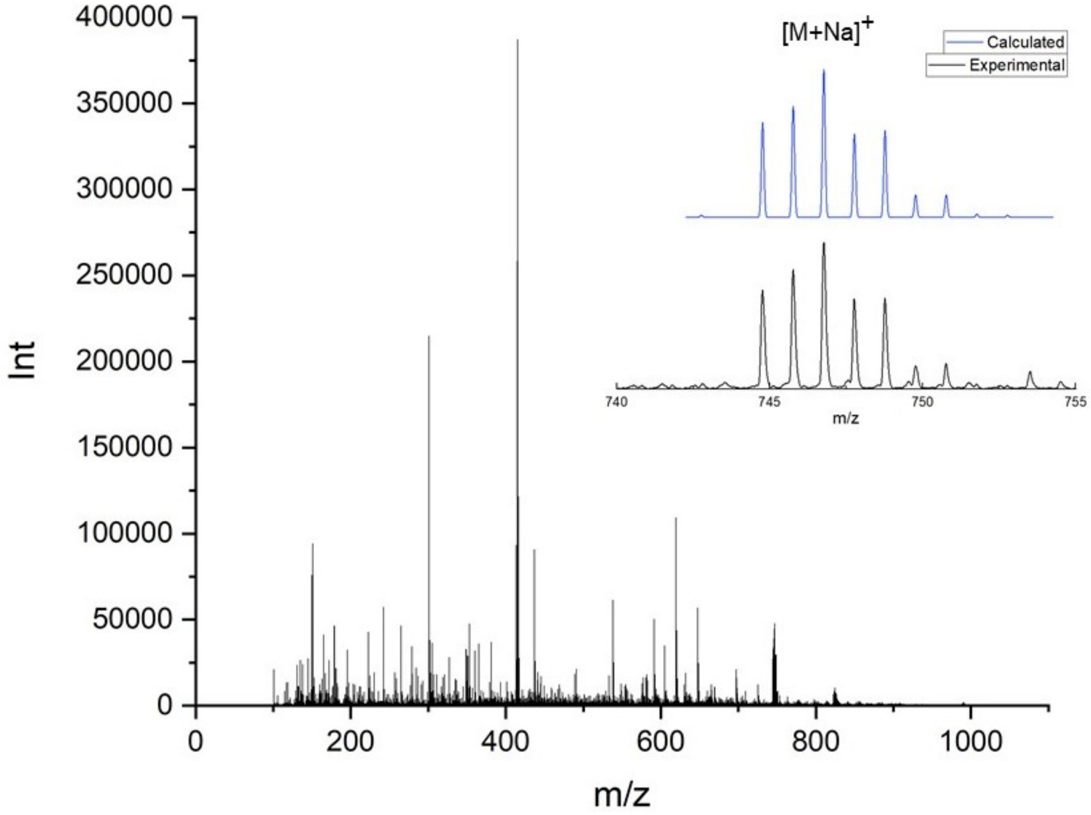

Figure S14. Mass-spectrum (ESI$^+$-HR) spectrum of *trans*-[$PtCl_2(2\text{-}ClC_6H_4CN)_2$].

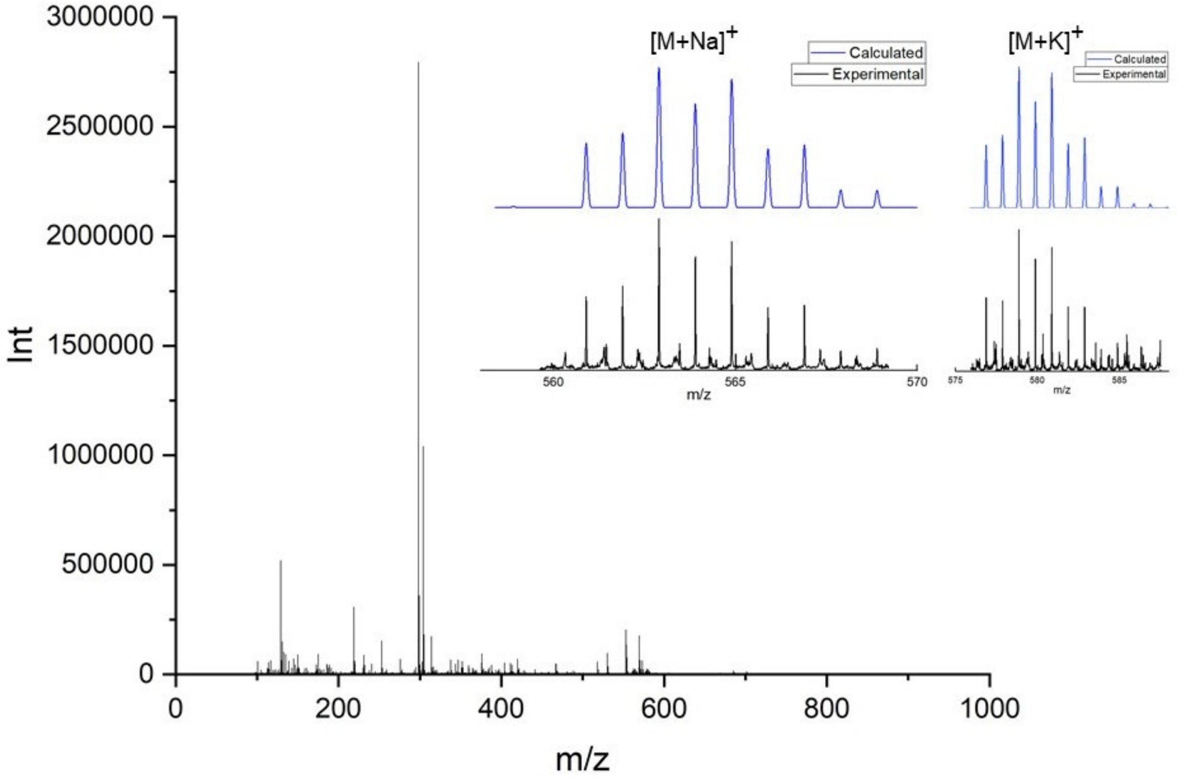

Figure S15. Mass-spectrum (ESI^{+}-HR) spectrum of **4**.

S5. The environment of complex **3** (Pt1A).

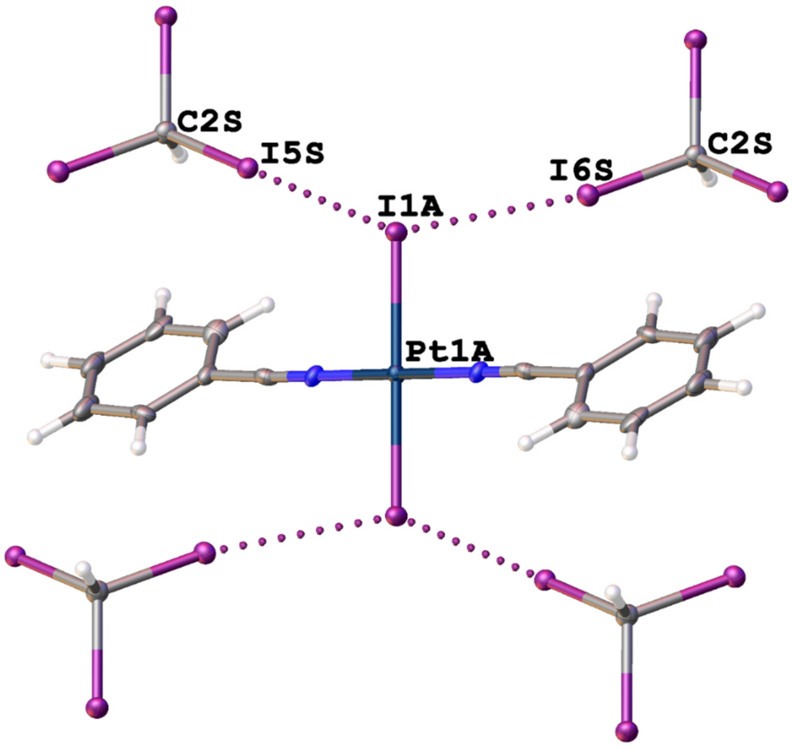

Figure S16. The environment of another crystallographically independent complex molecule in **3**·2CHI$_3$.

S6. Cartesian coordinates of theoretical models

Table S2. **1**·4I_2 (shifted 0.5c).

I	5.844079000	8.578369000	2.153017000
Pt	3.763932000	8.817500000	4.884118000
I	5.547449000	8.723682000	9.186440000
I	6.090837000	9.164204000	6.026709000
N	3.803734000	10.677993000	4.261882000
N	3.836946000	12.965252000	3.347575000
C	3.804454000	11.766072000	3.855523000
C	3.578955000	14.134453000	4.194481000
H	2.516708000	14.395451000	4.142709000
H	4.181043000	14.979169000	3.843801000
H	3.846920000	13.899907000	5.230891000
C	3.760404000	13.129258000	1.880386000
H	4.024751000	12.185785000	1.392951000
H	4.458138000	13.912252000	1.563895000
H	2.741439000	13.413181000	1.598084000
I	1.980415000	8.911318000	0.581796000
I	1.437027000	8.470796000	3.741528000
N	3.724130000	6.957008000	5.506355000
N	3.690919000	4.669748000	6.420662000
C	3.723410000	5.868928000	5.912714000
C	3.948910000	3.500548000	5.573756000
H	5.011156000	3.239550000	5.625527000
H	3.346821000	2.655831000	5.924435000
H	3.680944000	3.735093000	4.537346000
C	3.767460000	4.505743000	7.887851000
H	3.503113000	5.449215000	8.375286000
H	3.069726000	3.722749000	8.204342000
H	4.786426000	4.221819000	8.170153000
I	1.683785000	9.056631000	7.615219000

N	-0.039802000	1.860493000	0.622237000
N	-0.073013000	4.147752000	1.536544000
C	-0.040522000	2.948572000	1.028595000
C	0.184978000	5.316953000	0.689638000
H	1.247224000	5.577951000	0.741409000
C	0.003528000	4.311758000	3.003733000
H	1.022494000	4.595681000	3.286035000
I	1.783517000	0.093818000	4.302322000
I	1.455311000	0.239131000	7.037135000
Pt	0.000000000	17.635000000	0.000000000
I	2.326905000	17.288296000	1.142591000
H	0.082988000	12.552593000	0.346773000
H	-0.207725000	11.473331000	8.727919000
H	-0.364017000	14.266715000	6.277069000
H	0.069371000	12.540249000	6.448013000
H	7.735589000	6.161669000	1.040317000
H	7.891881000	3.368285000	3.491168000
H	7.458494000	5.094752000	3.320224000
I	5.200960000	0.346704000	8.625646000
H	7.444876000	5.082407000	9.421464000
I	5.744347000	17.541182000	5.465914000
I	6.072553000	17.395869000	2.731101000
N	7.567666000	15.774508000	9.146000000
N	7.600878000	13.487248000	8.231693000
C	7.568386000	14.686428000	8.739641000
C	7.342887000	12.318048000	9.078599000
H	6.280641000	12.057050000	9.026827000
C	7.524337000	13.323243000	6.764504000
H	6.505371000	13.039319000	6.482202000
I	3.526193000	2.873447000	1.519449000
I	4.417378000	5.449391000	1.897773000

I	0.237739000	11.690947000	3.364669000
I	7.499254000	14.266891000	2.986345000
I	4.001672000	14.761553000	8.248787000
I	3.110486000	12.185609000	7.870463000
I	0.028610000	3.368109000	6.781891000
I	7.290125000	5.944053000	6.403567000

Table S3. **2**·2CHI_3 (shifted 0.5b).

C	2.729877000	4.995404000	4.038333000
H	2.660646000	5.226815000	5.106262000
H	3.791166000	4.970355000	3.767718000
C	2.114081000	3.626436000	3.780432000
H	2.218207000	3.374208000	2.719768000
H	1.053164000	3.647684000	4.049230000
H	2.629612000	2.875034000	4.387045000
C	2.777394000	6.318064000	2.002367000
H	3.162292000	5.383657000	1.579191000
H	3.614313000	7.004050000	2.164917000
Pt	6.859712000	7.191817000	4.540514000
I	6.491007000	8.388906000	2.254547000
N	8.723549000	6.818562000	4.040149000
N	10.922948000	6.063866000	3.262813000
C	9.728836000	6.451994000	3.654206000
C	10.609638000	6.939358000	1.081550000
H	11.070781000	7.168267000	0.115329000
H	10.228910000	7.862428000	1.531969000
H	9.782832000	6.236265000	0.934438000
I	7.228416000	5.994728000	6.826481000
N	4.995874000	7.565072000	5.040879000
N	2.796476000	8.319768000	5.818215000
C	3.990588000	7.931640000	5.426822000
C	3.109785000	7.444276000	7.999477000
H	2.648642000	7.215366000	8.965699000
H	3.490514000	6.521206000	7.549058000
H	3.936591000	8.147368000	8.146590000
C	10.989546000	9.388230000	5.042695000
H	11.058777000	9.156818000	3.974766000
H	9.928258000	9.413278000	5.313309000

C	11.605343000	10.757198000	5.300596000
H	11.501216000	11.009426000	6.361260000
H	12.666259000	10.735949000	5.031798000
H	11.089811000	11.508599000	4.693983000
C	10.942029000	8.065569000	7.078661000
H	10.557132000	8.999977000	7.501837000
H	10.105111000	7.379583000	6.916111000
I	8.350783000	1.390619000	1.566386000
I	4.835465000	1.439447000	1.701058000
I	6.659256000	4.479651000	2.171728000
C	6.586422000	2.553848000	1.205960000
H	6.547127000	2.742522000	0.128042000
I	5.368640000	12.993015000	7.514641000
I	8.883959000	12.944186000	7.379970000
I	7.060168000	9.903983000	6.909300000
C	7.133002000	11.829785000	7.875067000
H	7.172296000	11.641111000	8.952985000

Table S4. **3**·2CHI_3 (shifted 0.5a).

C	3.812523000	0.299329000	5.373203000
H	3.876741000	0.381419000	4.293781000
C	3.322907000	1.291419000	7.511819000
H	3.017020000	2.152365000	8.096582000
C	3.599268000	0.107118000	8.153587000
H	3.520146000	0.022024000	9.233009000
C	3.428532000	1.408548000	6.136337000
H	3.215674000	2.355588000	5.650874000
Pt	4.962953000	5.005500000	9.194392000
I	2.476188000	4.893477000	9.964699000
N	4.595710000	6.780450000	8.486424000
C	4.308816000	7.801572000	8.015671000
C	4.111252000	9.097997000	5.998422000
H	4.434520000	8.235049000	5.426530000
C	3.978794000	9.037931000	7.379419000
I	7.449717000	5.117523000	8.424086000
N	5.330195000	3.230550000	9.902361000
C	5.617089000	2.209428000	10.373113000
C	5.814654000	0.913003000	12.390363000
H	5.491386000	1.775951000	12.962254000
C	5.947111000	0.973069000	11.009365000
C	6.113382000	9.711671000	13.015582000
H	6.049165000	9.629581000	14.095003000
C	6.602999000	8.719581000	10.876966000
H	6.908885000	7.858635000	10.292203000
C	6.326637000	9.903882000	10.235198000
H	6.405760000	9.988976000	9.155776000
C	6.497373000	8.602452000	12.252447000
H	6.710231000	7.655412000	12.737911000
N	7.819643000	1.774950000	0.707968000

C	8.106536000	2.796072000	1.178721000
C	8.304101000	4.092497000	3.195971000
H	7.980833000	3.229549000	3.767862000
C	8.436558000	4.032431000	1.814973000
C	8.602829000	5.304829000	3.821189000
H	8.538612000	5.386919000	4.900611000
C	9.092446000	6.296919000	1.682574000
H	9.398332000	7.157865000	1.097810000
C	8.816084000	5.112618000	1.040805000
C	8.986821000	6.414048000	3.058055000
H	9.199679000	7.361088000	3.543519000
I	4.965635000	0.112023000	0.770306000
H	3.916312000	5.027524000	18.350168000
Pt	7.452400000	10.011000000	0.000000000
H	6.009593000	4.983476000	0.038616000
I	4.960270000	9.898977000	17.618478000
N	2.106263000	8.236050000	17.680816000
C	1.819369000	7.214928000	17.210064000
C	1.621804000	5.918503000	15.192814000
H	1.945072000	6.781451000	14.620923000
C	1.489347000	5.978569000	16.573812000
C	1.323076000	4.706171000	14.567595000
H	1.387294000	4.624081000	13.488174000
C	0.833459000	3.714081000	16.706211000
H	0.527573000	2.853135000	17.290974000
C	1.109821000	4.898382000	17.347979000
C	0.939085000	3.596952000	15.330730000
H	0.726227000	2.649912000	14.845266000
N	-1.716702000	3.247568000	8.883622000
C	-1.251063000	2.222442000	8.688701000
C	-0.682019000	0.940033000	8.381608000

C	-0.046855000	0.776854000	7.142204000
H	0.051915000	1.608768000	6.452625000
H	-1.231609000	0.060066000	10.273814000
I	-1.229912000	5.126633000	11.463201000
C	-0.268498000	8.673530000	8.962694000
H	-0.341933000	7.842617000	9.657790000
C	0.446676000	9.550494000	6.827756000
H	0.928158000	9.392320000	5.869700000
C	-0.772478000	9.910890000	9.304725000
C	0.334871000	8.501341000	7.719612000
H	0.724540000	7.526270000	7.447458000
C	10.194404000	1.337470000	9.426091000
H	10.267839000	2.168383000	8.730995000
C	9.479230000	0.460506000	11.561029000
H	8.997748000	0.618680000	12.519085000
C	10.698384000	0.100110000	9.084060000
C	9.591034000	1.509659000	10.669173000
H	9.201365000	2.484730000	10.941327000
Pt	12.415353000	5.005500000	9.194392000
I	11.155817000	4.884367000	6.925584000
N	11.642608000	6.763432000	9.505163000
C	11.176968000	7.788558000	9.700084000
C	10.607924000	9.070967000	10.007177000
C	9.972760000	9.234146000	11.246581000
H	9.873990000	8.402232000	11.936160000
H	11.157515000	9.950934000	8.114971000
H	2.147514000	7.173883000	0.463397000
C	1.716969000	5.105610000	0.110333000
H	1.257838000	4.945434000	1.079422000
N	-4.206149000	1.757932000	18.078014000
C	-3.740510000	2.783058000	17.883093000

C	-3.171466000	4.065467000	17.576000000
C	-2.536302000	4.228646000	16.336596000
H	-2.437532000	3.396732000	15.647017000
C	-2.757945000	6.342970000	18.157086000
C	-2.042772000	5.466006000	16.022148000
H	-1.561290000	5.624180000	15.064092000
C	-2.154576000	6.515159000	16.914004000
H	-1.764907000	7.490230000	16.641850000
I	1.259535000	9.889867000	2.268808000
I	8.666370000	0.121133000	16.119976000
Pt	14.904800000	10.011000000	0.000000000
N	14.132055000	8.253068000	0.310770000
C	13.666416000	7.227942000	0.505692000
C	13.097372000	5.945533000	0.812784000
C	12.462208000	5.782354000	2.052188000
H	12.363437000	6.614268000	2.741768000
C	12.683851000	3.668030000	0.231699000
C	11.968677000	4.544994000	2.366637000
H	11.487195000	4.386820000	3.324692000
C	12.080482000	3.495841000	1.474781000
H	11.690812000	2.520770000	1.746935000
H	7.778392000	2.837117000	17.925387000
C	8.208936000	4.905390000	18.278452000
H	8.668067000	5.065566000	17.309363000
I	0.649881000	6.709172000	3.813650000
I	0.946084000	4.745614000	6.745374000
I	0.506147000	3.129238000	3.645393000
C	0.069759000	4.881364000	4.806828000
H	-1.016383000	4.916402000	4.942905000
I	11.765472000	1.703672000	5.380742000
I	11.469269000	9.751114000	2.449018000

I	11.909206000	8.134738000	5.549000000
C	12.345594000	9.886864000	4.387564000
H	13.431736000	9.921902000	4.251487000
I	9.276025000	3.301828000	14.575135000
I	8.979822000	5.265386000	11.643411000
I	9.419759000	6.881762000	14.743392000
C	9.856147000	5.129636000	13.581956000
H	10.942289000	5.094598000	13.445879000
I	-1.543363000	0.259886000	15.939766000
I	-1.983300000	1.876262000	12.839785000
C	-2.419689000	0.124136000	14.001221000
H	-3.505830000	0.089098000	14.137298000
I	-1.839566000	8.307328000	13.008042000
I	2.063771000	1.763338000	11.600749000
I	2.616265000	0.041045000	14.655494000
I	2.282630000	8.188998000	11.674304000
C	1.723089000	9.983970000	12.732394000
H	0.642893000	9.921902000	12.896055000
I	5.372687000	6.768838000	15.982428000
I	4.820193000	5.046545000	12.927683000
I	5.153828000	3.183498000	15.908873000
C	5.713370000	4.978470000	14.850782000
H	6.793565000	4.916402000	14.687122000
I	7.643275000	1.822002000	6.714481000
C	8.202817000	0.027030000	5.656390000
H	9.283013000	0.089098000	5.492730000
I	7.862135000	8.247662000	6.788036000
I	7.309640000	9.969955000	3.733291000
I	4.553218000	3.242162000	2.406356000
I	5.105712000	4.964455000	5.461101000
I	4.772077000	6.827502000	2.479911000

C	4.212536000	5.032530000	3.538002000
H	3.132340000	5.094598000	3.701662000

Table S5. **4**·4I$_2$.

C	6.914042	0.396644	1.667536
H	7.199566	0.825944	2.622633
C	6.185745	1.166143	0.760001
H	5.907799	2.185814	1.005569
Cl	3.383095	-2.998362	8.119679
C	4.299350	-3.912453	9.255894
C	5.774776	8.058693	0.150447
C	6.125565	8.604726	1.369554
H	6.683649	8.017489	2.090729
Pt	2.853052	2.495976	4.853132
I	5.250050	3.373337	4.311620
N	2.667379	1.698874	3.086592
C	2.480792	1.237395	2.059669
H	1.174575	2.485223	0.095121
C	2.173239	0.689478	0.760971
I	0.456055	1.618614	5.394645
N	3.038726	3.293077	6.619673
C	3.225313	3.754556	7.646596
H	4.531530	2.506728	9.611144
C	3.532866	4.302473	8.945294
C	-0.068671	-3.066742	9.555818
C	-0.419460	-3.612775	8.336711
H	-0.977544	-3.025538	7.615535
Cl	2.323010	7.990313	1.586586
C	1.406755	8.904405	0.450371
C	-1.207937	4.595307	8.038729
H	-1.493462	4.166007	7.083632
C	-0.479640	3.825808	8.946264
H	-0.201694	2.806137	8.700696
I	4.050700	4.500309	1.247255

I	1.655405	0.491642	8.459010
I	5.163894	-0.300103	5.068417
I	4.552410	6.560922	4.790721
I	1.153694	-1.568971	4.915544
I	0.542211	5.292054	4.637848
I	-0.235472	4.915758	1.309666
I	5.941576	0.076193	8.396599
